# Archaeological application of airborne LiDAR to examine social changes in the Ceibal region of the Maya lowlands

**DOI:** 10.1371/journal.pone.0191619

**Published:** 2018-02-21

**Authors:** Takeshi Inomata, Daniela Triadan, Flory Pinzón, Melissa Burham, José Luis Ranchos, Kazuo Aoyama, Tsuyoshi Haraguchi

**Affiliations:** 1 School of Anthropology, University of Arizona, Tucson, Arizona, United States of America; 2 Facultad de Ciencias Sociales, Universidad del Valle de Guatemala, Guatemala City, Guatemala; 3 Escuela de Historia, Universidad de San Carlos, Guatemala City, Guatemala; 4 Faculty of Humanities, Ibaraki University, Mito, Ibaraki, Japan; 5 Graduate School of Science Biology and Geosciences, Osaka City University, Osaka, Japan; New York State Museum, UNITED STATES

## Abstract

Although the application of LiDAR has made significant contributions to archaeology, LiDAR only provides a synchronic view of the current topography. An important challenge for researchers is to extract diachronic information over typically extensive LiDAR-surveyed areas in an efficient manner. By applying an architectural chronology obtained from intensive excavations at the site center and by complementing it with surface collection and test excavations in peripheral zones, we analyze LiDAR data over an area of 470 km^2^ to trace social changes through time in the Ceibal region, Guatemala, of the Maya lowlands. We refine estimates of structure counts and populations by applying commission and omission error rates calculated from the results of ground-truthing. Although the results of our study need to be tested and refined with additional research in the future, they provide an initial understanding of social processes over a wide area. Ceibal appears to have served as the only ceremonial complex in the region during the transition to sedentism at the beginning of the Middle Preclassic period (c. 1000 BC). As a more sedentary way of life was accepted during the late part of the Middle Preclassic period and the initial Late Preclassic period (600–300 BC), more ceremonial assemblages were constructed outside the Ceibal center, possibly symbolizing the local groups’ claim to surrounding agricultural lands. From the middle Late Preclassic to the initial Early Classic period (300 BC-AD 300), a significant number of pyramidal complexes were probably built. Their high concentration in the Ceibal center probably reflects increasing political centralization. After a demographic decline during the rest of the Early Classic period, the population in the Ceibal region reached the highest level during the Late and Terminal Classic periods, when dynastic rule was well established (AD 600–950).

## Introduction

Airborne LiDAR (Light Detection and Ranging) is rapidly becoming an important tool for archaeological research. As its laser pulses penetrate vegetation, LiDAR provides highly accurate and detailed three-dimensional maps of ground surface topography. The impacts of LiDAR are particularly significant in the Maya lowlands and other tropical regions, where dense vegetation has prevented archaeologists from conducting extensive surveys [1–11]. In those areas, traditional investigations have typically focused on the site centers and covered small sample areas in peripheral zones through pedestrian surveys of quadrants or transects. LiDAR provides continuous spatial data over wide areas on the distribution of archaeological remains, natural topography, vegetation types, and other environmental conditions.

As revolutionary as LiDAR is, a remaining challenge is that it only presents a synchronic view of modern terrains and does not offer temporal information directly [12,13]. In traditional ground surveys, researchers commonly conduct surface observations, surface collections, and test pits, along with mapping, to obtain chronological data on regional scales. Although LiDAR typically offers continuous spatial data in broader areas than pedestrian surveys, conducting surface collection and test excavations over the entire LiDAR-surveyed areas would require many years of additional work. While data from extensive surface collection and excavations continue to be critical, we also need to develop methods to extrapolate diachronic patterns over wide regions efficiently and logically from LiDAR and available archaeological data. As areas covered by LiDAR are rapidly expanding and nation-wide LiDAR data may soon become available in various parts of the world, the importance of this methodological issue will only increase in the near future [14].

Scholarly discussion on this issue is still at the beginning stage, and much work needs to be done. In our view, strategies for this question should consider the following points while specific methods need to be refined for the settings of individual study areas, including natural topography, the nature of archaeological features, and available archaeological information. First, when possible, the morphological data on sites and features readily available from LiDAR data should be utilized for the understanding of diachronic processes. In this regard, the development of architectural chronologies is particularly effective for the Maya lowlands, where a significant portion of archaeological remains made of stone and earth are visible on the ground surface. Maya archaeologists have long discussed the architectural chronology of residential buildings and ceremonial complexes [15–17]. Researchers need to refine an architectural chronology by combining LiDAR data with existing archaeological information, additional surface collection, and excavation. Second, we need to evaluate the effectiveness of LiDAR rigorously, particularly, in relation to vegetation types. Many scholars have noted that the effectiveness of LiDAR is affected significantly by land cover [18–21]. Nonetheless, the effects of vegetation types are not always evaluated systematically in archaeological research. It is necessary to classify vegetation types by analyzing LiDAR and other available remote sensing data, and to assess the detection rates of archaeological features in each vegetation type through ground-truthing [22]. Such evaluations should address both false positives (commission errors; erroneous identifications of natural or modern features as archaeological ones) and false negatives (omission errors; archaeological features not identified in LiDAR data).

Third, the results of ground-truthing, surface collection, and excavation should be logically incorporated in the interpretation of overall settlement patterns. Strategies for ground-truthing and archaeological data collection need to be developed according to the local conditions and objectives of the research. In this respect, researchers have employed various methods, including comparison with existing archaeological maps [1], surface collection at all identified archaeological features [23], and the use of survey quadrangles for ground-truthing [24]. Settlement data and associated archaeological information are inevitably incomplete because it is impossible to excavate entire archaeological sites or regions. The key is to develop logical processes for extrapolation. In the Maya lowlands, where vegetation is dense and access to private lands is often difficult, archaeologists have routinely applied sampling survey methods even before the use of LiDAR. For LiDAR-based settlement data in tropical region, ground-truthing should sample different vegetation types. Commission and omission errors for each vegetation type should then be incorporated in the interpretation of overall patterns.

In this study, we obtained LiDAR data for an area of 470 km^2^ around the Maya site of Ceibal, Guatemala (16° 30’ 49” N 90° 3’ 41” W), in 2015. Prior to the LiDAR survey, we had been conducting intensive excavations in the Ceibal site center, through which we established a detailed chronology of the site and refined the ceramic sequence. During the 2016 and 2017 field seasons, we ground-truthed sample areas within the LiDAR-surveyed region and carried out surface collections of archaeological materials. In addition, we conducted small test excavations at select sites to obtain better chronological data. The main goal of this study was to examine the process of social change in the Ceibal region from the beginning of sedentary occupation around 1000 BC to the abandonment of the region around AD 950. For this purpose, we developed an architectural chronology based on the intensive excavations at the site center. Data obtained from surface collection and test excavations outside the center served to verify the applicability of this architectural chronology to the entire LiDAR-surveyed area. The results of vegetation classification and ground-truthing were discussed in detail in our previous publication [22]. This paper focuses on the interpretation of the LiDAR data and the reconstruction of diachronic patterns.

## Background

### Previous studies

**Fig 1:**
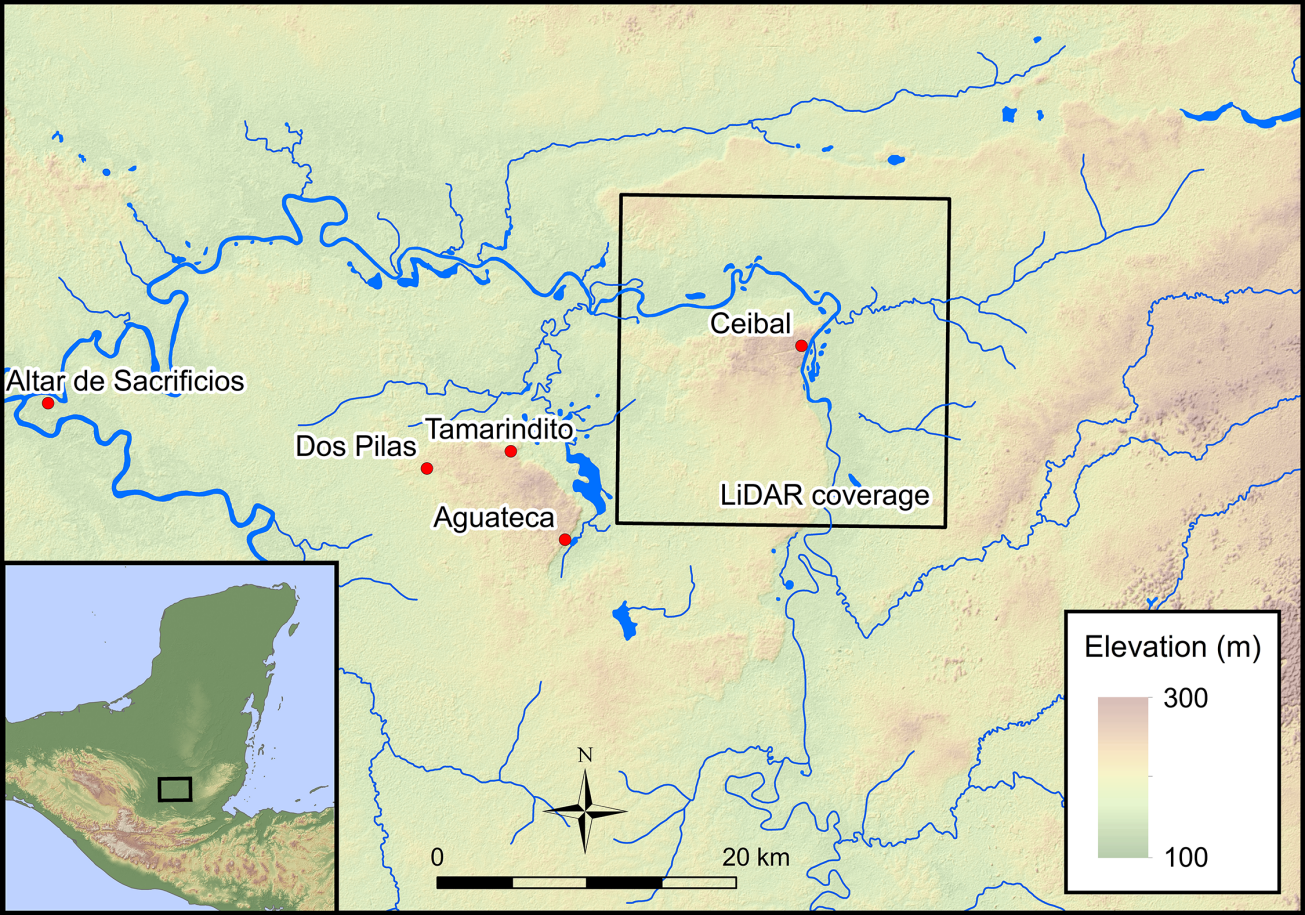
Map of the Pasión River region. It shows the locations of archaeological sites and the extent of the LiDAR survey. Topographic data from the NASA Shuttle Radar Topography Mission (SRTM; https://lta.cr.usgs.gov/SRTM).

**Fig 2:**
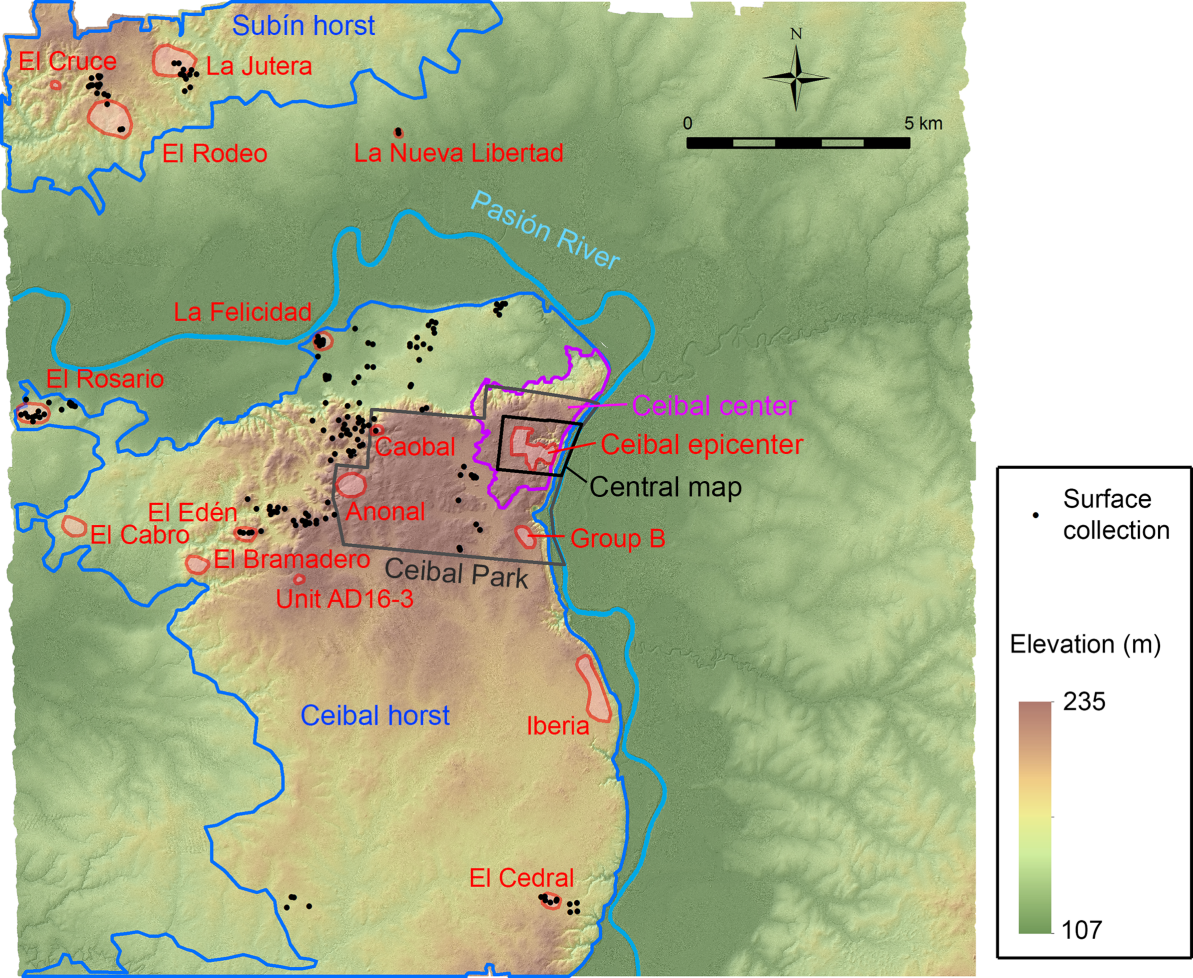
Map of the LiDAR-surveyed arear around Ceibal. It shows the locations of surface collection, groups, sites, and zones mentioned in the text.

Ceibal is the largest site in the Pasión River region of the southwestern Maya lowlands, located above a steep escarpment overlooking the Pasión River (Figs 1 and 2). The site was originally investigated by Harvard University from 1964 to 1968 under the direction of Gordon Willey, which represents a landmark study in the history of Maya archaeology. Harvard researchers completely mapped an area of 1.9 km^2^ in the Ceibal site center and extensively excavated multiple buildings [25–27]. Jeremy Sabloff developed a ceramic chronology of the site, which served as a basis for later chronological refinements [28]. Gair Tourtellot surveyed an area of roughly 6 km^2^ beyond the site center by using survey transects and conducted test excavations of select groups [15]. His architectural typology provided significant reference data for our assessment of architectural chronology in the LiDAR-surveyed area.

**Fig 3:**
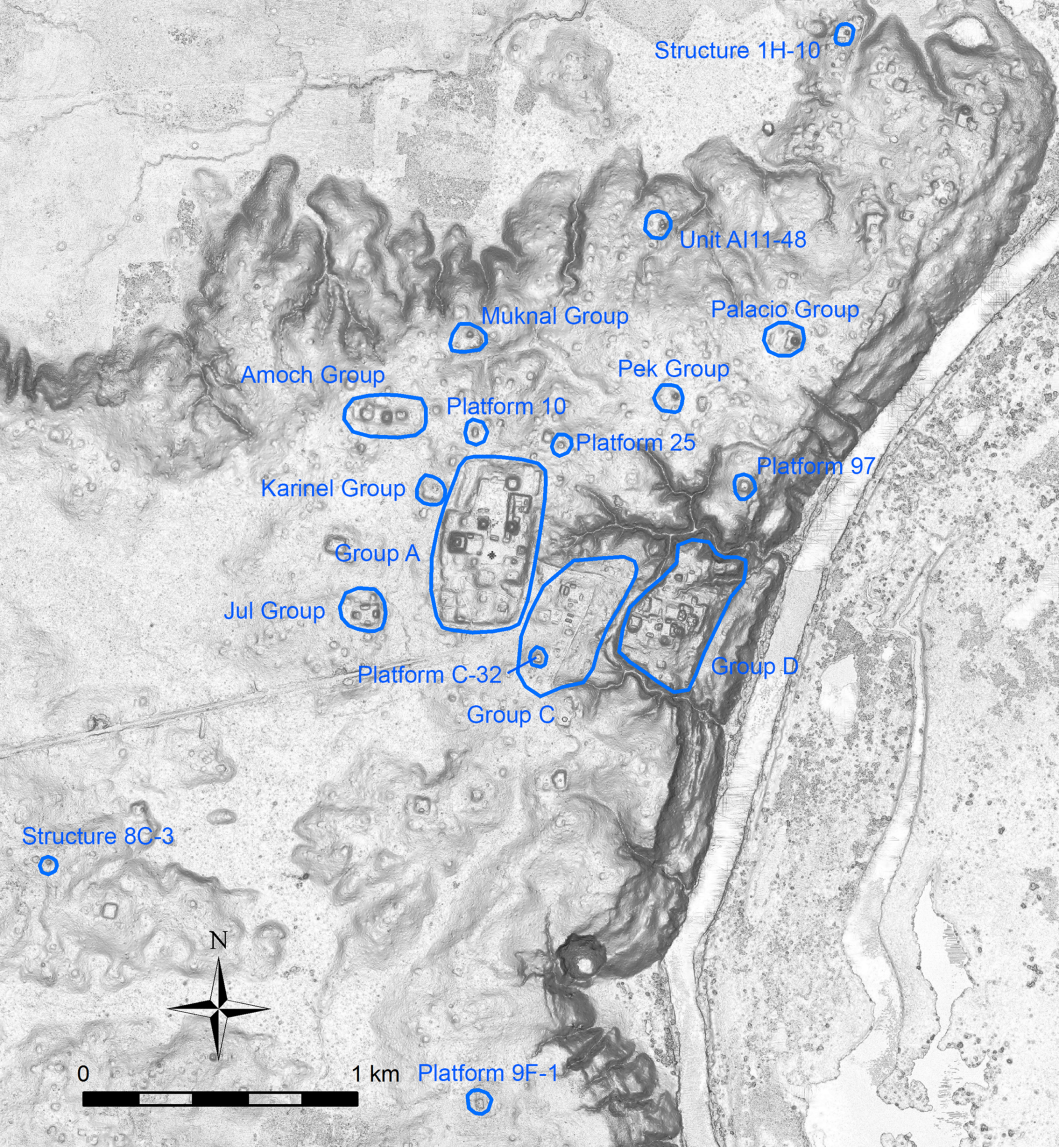
Map of the Ceibal center. It shows the locations of the groups, structures, and platforms mentioned in the text.

**Fig 4:**
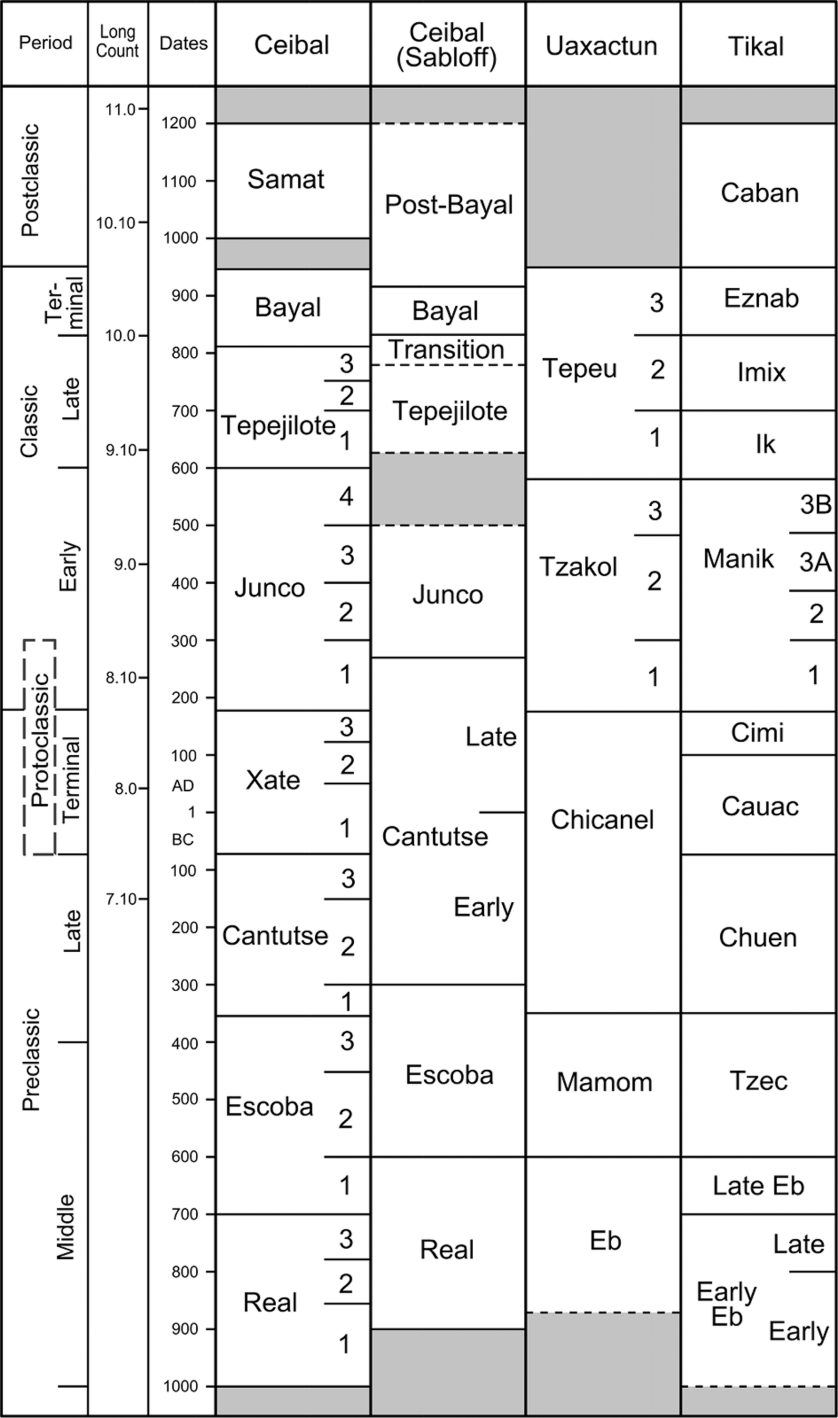
Chronology of Ceibal and other Maya sites.

In 2005 we initiated the Ceibal-Petexbatun Archaeological Project (CPAP) to revisit this important site. We conducted a series of deep stratigraphic excavations in the core area of Ceibal called Group A to document initial constructions and to refine the chronology of the site. We also carried out smaller-scale excavations in residential groups and temple complexes outside Group A [29–33] (Fig 3). In addition, Jessica Munson excavated the minor center of Caobal, located 4 km west of Group A [34,35]. With 154 radiocarbon dates, detailed ceramic analysis, and stratigraphic information, we subdivided the ceramic phases established by Sabloff into multiple facets [36,37] (Fig 4). We documented the beginning of ceramic use and sedentary occupation around 1000 BC and the expansion of a formal ceremonial complex during the Middle Preclassic period (1000–350 BC). This complex shared a standardized spatial configuration called the Middle Formative Chiapas (MFC) pattern with contemporaneous centers in Chiapas, which consisted of an E-Group assemblage (a square building on the west side and an elongated platform on the east side of an open plaza space) in the center and large platforms along the north-south axis of the E Group [37,38].

Although the Ceibal community grew extensively during the following Late Preclassic period (350–75 BC), the population declined during the Terminal Preclassic (75 BC-AD 175), and a substantial part of Ceibal was abandoned in the early part of the Early Classic (AD 175–600). A new dynasty was established at the beginning of the fifth century AD, but the population of Ceibal remained low throughout the Early Classic. At the beginning of the Late Classic period (AD 600–810), the population of Ceibal increased rapidly, but after a military defeat by the Dos Pilas-Aguateca dynasty in AD 735, construction activities declined. Ceibal experienced disruptions in dynastic rule around AD 770 and 810 amid the social upheaval generally called the Classic Maya collapse. With the return of its dynasty in AD 829, Ceibal had a brief revival during the Terminal Classic period (AD 810–950), but the center was completely abandoned shortly after AD 900. The area was occupied only occasionally by small groups during the Postclassic period (AD 1000–1200) [39–42].

### LiDAR survey

Here we briefly summarize the procedures of LiDAR data acquisition, archaeological feature identification, vegetation classification, and ground-truthing, which were discussed in detail in an earlier publication [22]. LiDAR data were obtained from March 18th to 23rd, 2015 by the crew of the National Center for Airborne Laser Mapping (NCALM) of the University of Houston, and a digital elevation model (DEM, bare earth model after the removal of vegetation and buildings) was produced at a horizontal resolution of 0.5 m. The NCALM crew collected most data from a flying altitude of 700 m above the ground level (AGL) and at a total pulse repetition frequency (PRF) of 450 kHz (150 kHz per channel for the three channels of Titan LiDAR), but they also used a total PRF of 750 kHz for some flight lines. The team also conducted canopy penetration tests over the central part of Ceibal with multiple settings, including 700 m AGL and 300 kHz total PRF, 600 m and 450 kHz, and 400 m and 150 kHz [43]. The canopy penetration test flights resulted in 51 to 72 laser shots per m^2^, whereas regular mapping flight lines produced 15 to 19 shots per m^2^. Ground point densities vary widely by vegetation type, ranging from 2.84 to 42.84 points/m^2^ for the test flight area and 0.56 to 12.79 points/m^2^ for other areas [22]. LiDAR covered a nominal survey polygon of 400 km^2^, and the addition of areas along the edges with reduced swath overlaps resulted in a total surveyed area of 470 km^2^.

**Fig 5:**
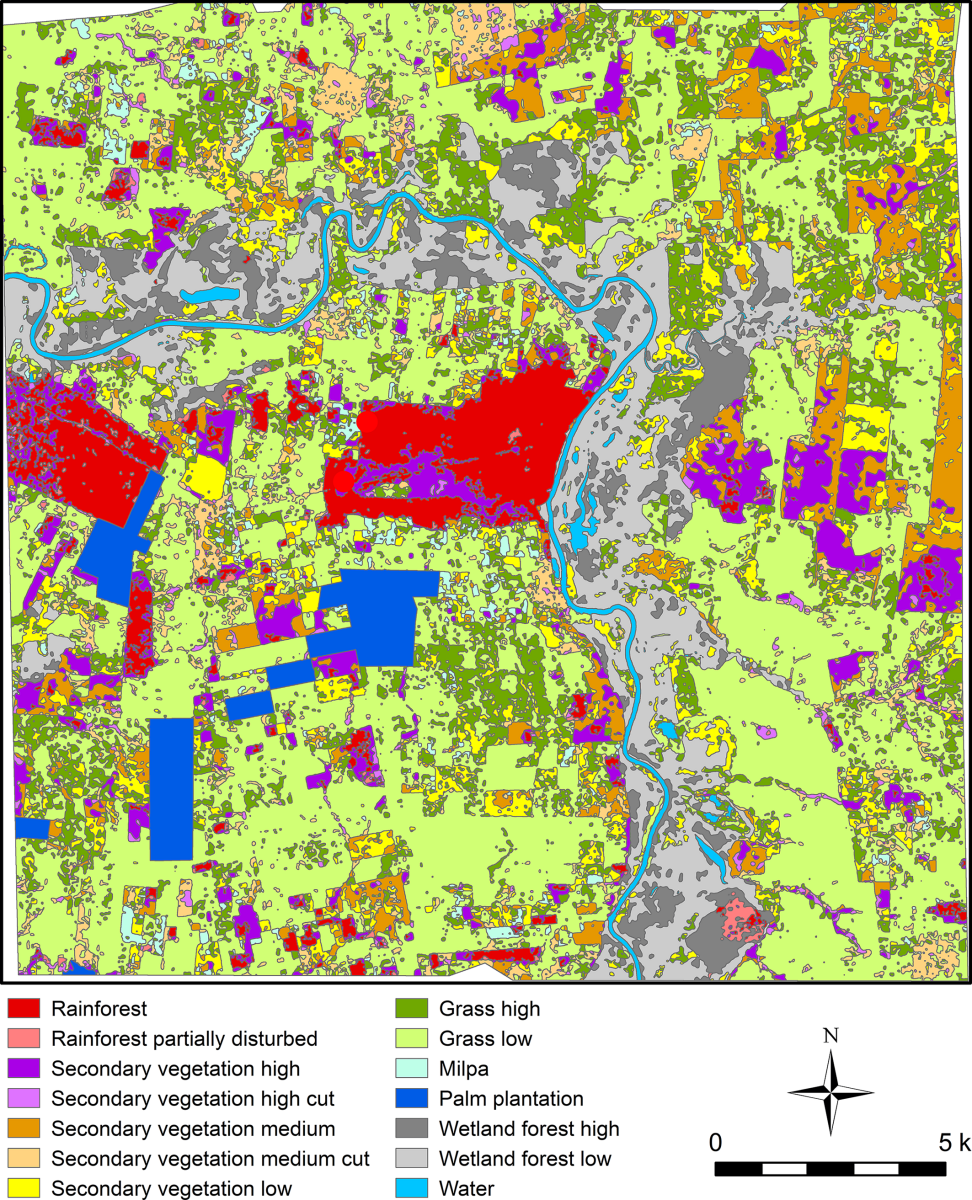
Vegetation classification based on the LiDAR data. See [22] for detailed discussion of its procedure.

We primarily used the Red Relief Image Map (RRIM) visualization of the LiDAR-derived DEM to identify archaeological features. RRIM tends to highlight subtle cultural remains, as well as larger-scale natural topography, better than other visualization techniques [44]. We also compared the RRIM with other images, including hillshades, elevation profiles, and LiDAR point cloud profiles when necessary, for better interpretations of archaeological features. In addition to the identification of archaeological features, LiDAR data also served for the classification of vegetation. The area around Ceibal presents diverse vegetation, including rainforest in the protected Ceibal Park, secondary vegetation in various stages, palm plantations, and pastures (Fig 5). The penetration rate of laser pulses, and thus the fidelity of the LiDAR-derived DEM, varies depending on the types of land cover. It is necessary to classify vegetation and to evaluate the effectiveness of LiDAR for the detection of archaeological features for each class. We conducted object-based image analysis (OBIA) for this purpose [22].

We ground-truthed archaeological features identified in the LiDAR data from February 7th to March 9th, 2016. While ground-truthing, the survey crew also collected archaeological artifacts found on the surface. In addition, during the 2016 and 2017 seasons, we conducted test excavations at two sites that were newly found in the LiDAR image (La Felicidad and El Edén) (Fig 2). In the central part of Ceibal, we identified additional structures that were not recorded on the Harvard map, but we focused our effort on ground verification outside of Ceibal to obtain archaeological information on previously-unknown sites. Instead of using survey transects or quadrangles, we purposefully selected target areas based on the LiDAR data. A reason for this approach was that it was often difficult to obtain permissions from landowners, and we had to adjust our survey strategy as needed. This social condition is shaped by the recent history of Guatemala and the region, including the civil war, ongoing drug-trafficking, conflicts over land rights involving violent clashes between police and squatters, and environmental feuds concerning oil palm plantations. The distribution of our ground-truthing and surface collection shown in Fig 2, including the absence of survey locations in the central part of the Ceibal horst, was largely conditioned by the availability of landowners’ permission.

Given these restrictions, our survey strategy had the following main goals. First, our important targets were minor centers, particularly E-Group assemblages, that were newly found in the LiDAR-surveyed area. We tried to visit as many of them as possible within the areas where we could obtain landowners’ permission. Second, we selected survey areas from different vegetation types to calculate commission and omission error rates for each land cover type. To obtain better data on omission errors, the survey crew also conducted systematic surveys of three areas of pasture, measuring 100 x 200 m to 130 x 400 m, by walking at regular intervals. It is desirable to conduct a systematic survey of sample areas for other vegetation types. Nonetheless, systematic coverage of densely vegetated areas requires the clearing of some vegetation, and we were not able to obtain landowners’ permission for clearing. Third, to test the applicability of the architectural chronology based primarily on data from the Ceibal center, we obtained samples of surface collection at varying distances from Ceibal. They include: vicinities of the Ceibal center; areas of mid distances (3 to 7 km from Group A), such as those around La Felicidad, Caobal, and El Edén; and distant areas (7 to 13 km from Group A), encompassing the Subín horst, El Rosario, and the southern portion of the Ceibal horst. Fourth, we assumed that another factor affecting the settlement distribution was the distance from the Pasión River. We thus conducted surface survey at varying distances from the river.

Field data showed that in nearly ideal conditions, such as low pastures, LiDAR effectively detected small structures measuring 10 to 30 cm in height. A substantial portion of archaeological features were identified in rainforest and 12-to-30-year-old high secondary vegetation. Nonetheless, because of the dense forest caused by precipitations (roughly 1800 mm) higher than those in the central Maya lowlands, laser penetration rates in some parts were low [19], causing misidentifications of features. As suggested by various scholars [20,21], young low secondary vegetation and high grasses blocked a substantial portion of laser pulses and created misclassified ground points. Survey of these areas would require substantial clearing of vegetation, but we were not able to obtain permission from landowners for such operations. Thus, we estimate that the rates of misidentifications in those areas are substantially higher than what our ground-truthing data indicate.

### Definitions of zones and archaeological features

The LiDAR data allow us to define zones of settlement distributions in the survey area, which can be used as frameworks for our analysis (Fig 2, Table 1). The Ceibal epicenter is the elite and ceremonial core of the site, consisting of Groups A, C, and D. The Ceibal center includes the epicenter and the surrounding densely-occupied residential zones, which are demarcated by escarpments to the north and east and by low, seasonally wet areas to the west and south. We define these zones as heuristic bases for our analysis, and they are not meant to represent political boundaries or patterns of social organization. The Ceibal horst is primarily a geological zone defined as an upland above the escarpment along the Pasión River, though its edges generally follow the limits of the dense distribution of archaeological features. Likewise, the Subín horst refers to the upland north of the Pasión River, a portion of which was covered by LiDAR.

**Table 1:** Archaeological zones in the Ceibal region.

Zone	Note	Area (km^2^)
Ceibal epicenter	Ceremonial core with Groups A, C, and D	0.49
Ceibal center	Area of dense occupation, consisting of the Ceibal epicenter and residential zones	5.41
Ceibal horst	Upland where most archaeological features are distributed	133.10
Subín horst	Upland in the northern part with dense archaeological features	27.02
LiDAR vegetation analysis zone	LiDAR area excluding edges where there are not sufficient flight swath overlaps	441.34
Total LiDAR area	Entire area covered by LiDAR	470.19

We define the categories of archaeological features in the following manner. A structure is an individual building, generally assumed to be a single roofed area, including residences, temples, kitchens, and workshops. A supporting platform refers to a raised building with an ample summit, which potentially supported multiple structures. Following Tourtellot [15], we define a unit as a group of structures, often surrounding a patio. Some units, however, consist of single structures, single platforms, or structures not surrounding formal patios. Terraces are artificially-built leveled spaces with retaining walls placed on natural slopes, which may have served as agricultural fields or residential areas. In addition, we plotted causeways (raised or paved streets), walls (elongated, unroofed constructions which potentially served for defense or property divisions), and depressions (artificial features such as reservoirs, storage pits, and quarries, as well as natural features that may have been used by humans, such as caves and sinkholes), although we do not discuss them in this paper. Within the categories of structures and supporting platforms, those that were reasonably identifiable were classified as “structures” and “supporting platforms,” while those that were difficult to distinguish from natural features or modern constructions were labeled as “possible structures” and “possible supporting platforms.” Within the 470 km^2^ area covered by LiDAR, we registered 10,208 structures, 4,538 possible structures, 724 supporting platforms, and 253 possible supporting platforms [22].

## Architectural chronology

**Fig 6:**
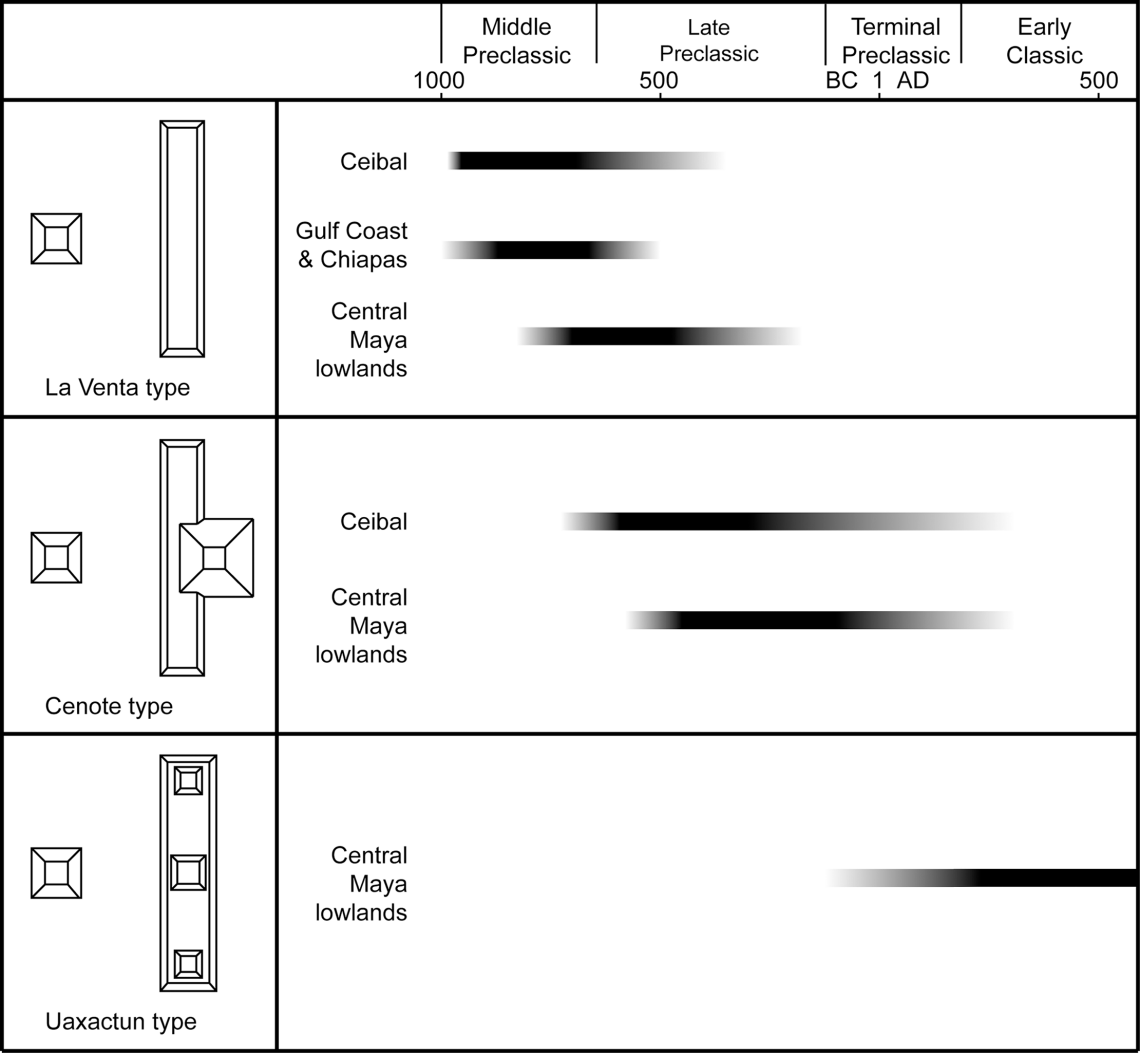
Chronology of E-Group assemblages. Whereas the beginning of each E-Group type can be determined based on archaeological evidence, its end point is represented rather arbitrarily. This is because some complexes continued to be used in their original E-Group configurations while their meaning and use pattern likely changed gradually, and new complexes were built in different formats.

In terms of architectural chronology, important configurations include E-Group assemblages, supporting platforms, and patio groups. E-Group assemblages are ceremonial complexes that spread throughout the Maya lowlands, as well as central Chiapas and some parts of the southern Gulf Coast, during the Preclassic period. Their arrangement with a western pyramid and an eastern long building may have been associated with symbolisms and rituals tied to solar movements [45–51]. Our work in the Ceibal region has provided significant information regarding the development of these groups, refining earlier chronologies [45,48] (Fig 6).

The earliest form dating to the Middle Preclassic period (1000–350 BC) included a relatively simple configuration of the eastern platform in a flat and linear rectangular shape. This configuration may be called the La Venta type, referring to Mounds D-1 and D-8 at this Olmec center [52,53]. The Late Preclassic period (350–75 BC) witnessed the development of what Arlen Chase has named the Cenote type, which is characterized by an eastern winged pyramid (the eastern platform supported a tall pyramid in the center) [45,54]. Single eastern winged pyramids that were not paired with a western structure, thus not forming an E Group, also emerged during this period. The well-known Uaxactun-type of E Group, with three buildings placed on the eastern platform, probably developed during the Terminal Preclassic and Early Classic periods in other parts of the Maya lowlands. This type, however, never gained popularity in the Ceibal region. New pyramidal constructions, possibly dating to the late part of the Late Preclassic period or later in the Ceibal region, did not take the forms of an E Group or eastern winged pyramid. In terms of residential complexes, Preclassic ones are characterized by supporting platforms, although residential groups without supporting platforms also existed. Many Classic-period groups consisted of individual structures, commonly surrounding patios.

All excavated materials are stored at the National Museum of Archaeology and Ethnology and the Salon 3 storage facility of the Guatemalan government (7a Avenida y 6a Calle, Zona 13, Guatemala City). Researchers interested in those materials should request permission from the Instituto de Antropología e Historia de Guatemala for access to these facilities (demopre.secre@gmail.com; http://mcd.gob.gt/tag/idaeh/).

### Excavations prior to the LiDAR survey

#### Preclassic architecture

**Fig 7:**
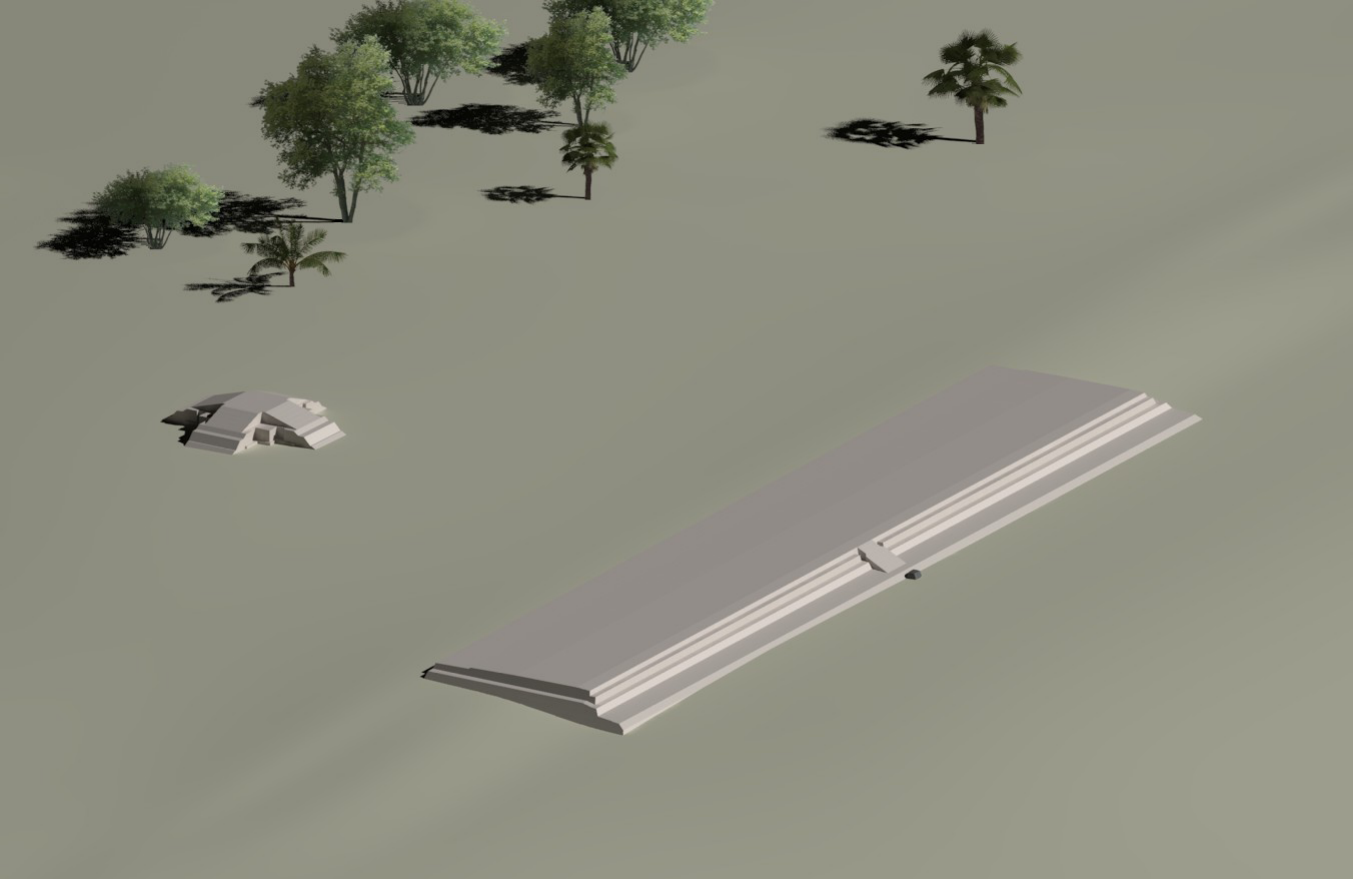
Reconstruction image of the earliest version of the E-Group assemblage at Ceibal. It represents a La Venta-type E Group, with a linear and flat eastern platform.

**Fig 8:**
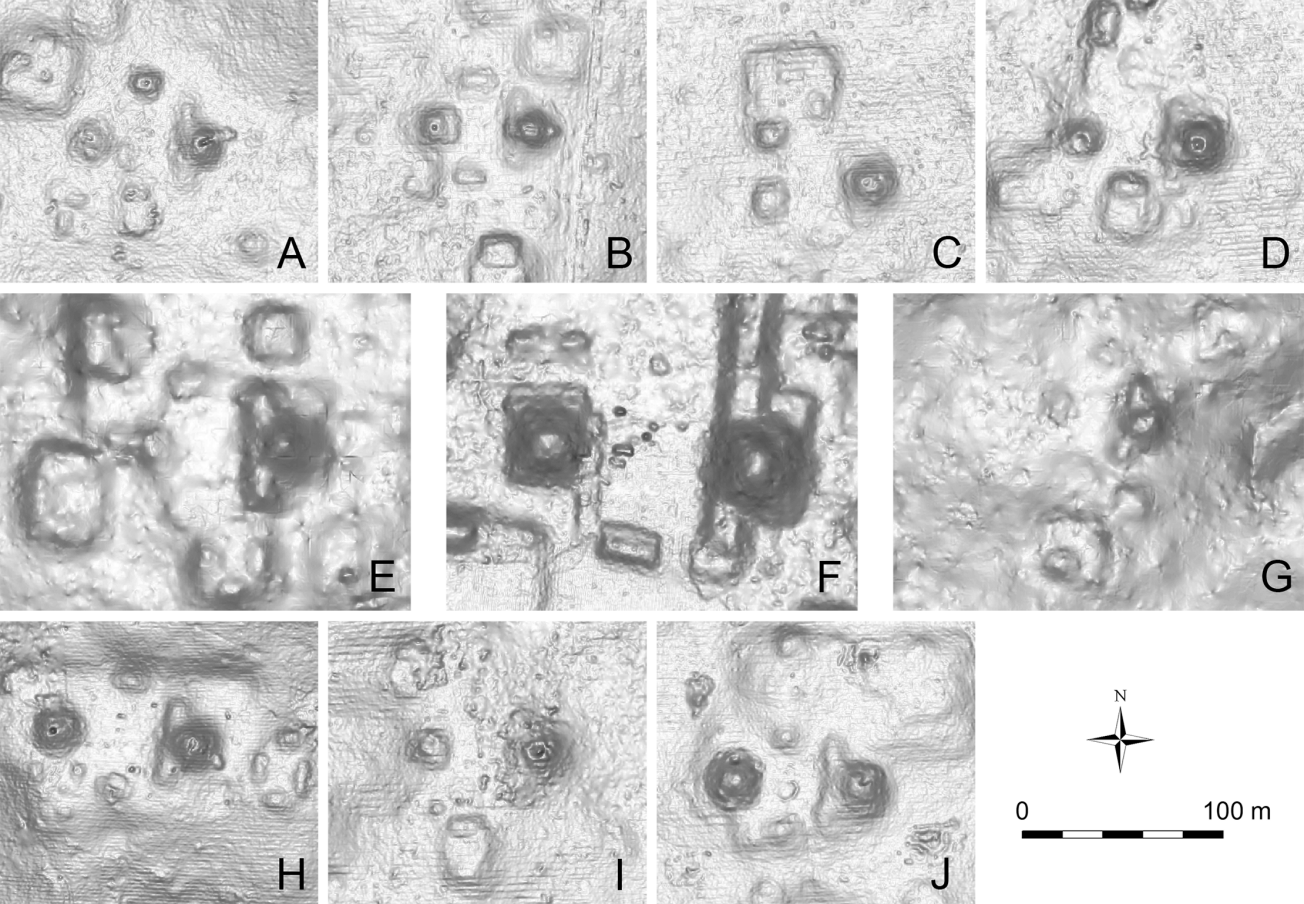
LiDAR images of the E-Group assemblages in the Ceibal region. (A) El Cruce. (B) El Rodeo. (C) La Nueva Libertad. (D) La Felicidad. (E) Anonal. (F) Ceibal Group A. (G) Unit AI11-48. (H) El Edén. (I) Unit AD16-3. (J) Iberia. Note that all the E-Group assemblages are of the Cenote type in their final forms. While the Group A complex is the largest and the one at Anonal is the second, all others are of similar sizes. In the E Groups located outside of the protected Ceibal Park, looters’ pits are visible.

The earliest example of E-Group assemblages, dating to c. 950 BC in the early Middle Preclassic period, was identified in Group A of Ceibal [29,31,37] (Figs 3 and 7). In its earliest form, the western building (Structure Ajaw) was still low, measuring 2.0 m in height. The eastern building (Structure Xa’an) appears to have had a simple, long rectangular form with a flat top, and did not appear to support any structures. Thus, this complex formed a La Venta-type E Group. During the late part of the early Middle Preclassic (775–700 BC), the plaza between the two buildings was expanded, and a new version of the eastern building (Structure Saqpusin) was built to the east of Structure Xa’an, but it retained the form of the La Venta type. The E-Group plaza was amplified again during the late Middle Preclassic Escoba 3 phase (450–350 BC), and the third version of the eastern building was constructed further east. The early constructions of this structure was excavated only in small parts, and it is not clear whether it had a simple shape like its predecessors or whether a central pyramid was added. By the end of the Late Preclassic Cantutse 1 phase (350–300 BC), this eastern structure was certainly equipped with a central pyramidal building (Structure A-10), while its northern and southern sections continued to be mostly flat. This observation confirms its shape of a winged pyramid that formed a Cenote-type E Group (Fig 8).

Another Cenote-type E-Group assemblage at the minor center of Anonal was excavated by the Harvard Project [15]. In a small test excavation in the plaza in front of the eastern pyramid, excavators revealed a series of construction layers dating to the late Middle Preclassic period on top of bedrock. It is not clear whether or not Anonal originally had a La Venta-type E Group.

Excavations of the Pek Group (Structures 4G-4, 4G-2a, 4G-2b, and 4G-3) indicate that its eastern winged pyramid, which does not have an associated western pyramid, was built around the same time period as the Cenote-type E Group of Group A. Although the excavation did not reach the core part of the winged pyramid, the earliest construction layer in the front part of the pyramid dates to the Late Preclassic Cantutse 1 phase (350–300 BC). A somewhat earlier development of a winged pyramid was found at the minor center of Caobal (Unit AE12-18). Excavations by Jessica Munson showed that this area was a small village, possibly established in the early Middle Preclassic Real 2 phase (850–775 BC). During the late Middle Preclassic Escoba 2 phase (600–450 BC), a possible winged pyramid was built, covering an earlier round or apsidal platform, transforming this area into a ceremonial complex [34,35]. These results demonstrate that in the Ceibal region, the earlier form of E Group with a linear platform with a flat summit was gradually replaced during the late Middle Preclassic and Late Preclassic periods by the Cenote type. Ceremonial complexes with an eastern winged pyramid and without a western pyramid also began to emerge around that time.

The mid-to-late part of the Late Preclassic period (300–75 BC) may be characterized by the emergence of pyramids that do not form E Groups or eastern winged pyramids. This new development is suggested by the results of excavations at the Amoch and Muknal Groups. The Amoch Group consists of a single pyramid (Structure 1) associated with two large platforms. The form of the main building at the Muknal Group (Unit 4E-10) is similar to that of a winged pyramid, but faces either north or south. Although excavations of the Muknal and Amoch Groups did not reach the core parts of the pyramids, the earliest layers of the frontal part and plaza of Structure 1 in the Amoch Group date to the Late Preclassic Cantutse 2 or 3 phase, and the earliest layer of the plaza in the Muknal Group dates to the Cantutse 3 phase. In addition, excavations by the Harvard Project dated a single-standing pyramidal structure, 8C-3, to the generic Preclassic period (likely the Late or Terminal Preclassic), and the lowest plaza layer in front of pyramidal structure 1H-10 to the Early Classic period [15] (Fig 3). We should note that the configurations of these pyramids are less characteristic than E-Group assemblages, and it is necessary to verify their chronological positions with more excavations.

If these late dates of non-E-Group pyramids are correct, they imply that the popularity of the E-Group format was declining at Ceibal around this time. This trend can also be seen in a change in the use of space in Group A. Toward the end of the Middle Preclassic period, the deposition of caches with greenstone axes along the central axis of the E Group ceased, and new ritual practices involved the caching of ceramic vessels, obsidian artifacts, and sacrificed individuals [31,55–57]. In addition, the supporting platforms located to the southwest and northeast of the E-Group assemblage (A-24 Platform and the East Court), which had held multiple buildings, were converted to flat, open spaces at the beginning of the Late Preclassic period. Although the form of the MFC pattern with an E Group persisted and their buildings continued to be renovated, the Ceibal residents appear to have substantially changed their use pattern and symbolism. Moreover, other E-Group assemblages in the Ceibal region, possibly built during the Late Preclassic period, did not clearly follow the format of the MFC pattern.

We can see a similar trend in E-Group configurations in other parts of southern Mesoamerica, though with some variations in timing. The presence of La Venta-type E Groups during the Middle Preclassic period is seen at Finca Acapulco, which is probably the earliest center in the Grijalva River region of Chiapas, as well as at La Venta [52,53,58]. Similarly, the E-Group assemblages at Cival and Tikal, the two earliest known examples in the Maya lowlands after that of Ceibal, had simple linear eastern platforms of the La Venta type during the Middle Preclassic period [59–61]. It is likely that this format defined early E Groups of southern Mesoamerica. This form was then replaced by the Cenote type of E Group with an eastern winged pyramid, but the timing of their emergence varied from the late Middle Preclassic to the Terminal Preclassic. A Cenote-type E Group may have been built during the Late Preclassic period at Uaxactun, but the eastern platforms with flat summits persisted for most part of the Late Preclassic period at Tikal and Caracol [17,45,59,62]. A potentially earlier example of the eastern winged pyramid or the Cenote-type E Group is Group E of Xunantunich in Belize, which may date to the late Middle Preclassic or even early Middle Preclassic [63]. The overall configuration of this complex, however, is not clear, and it is not certain whether it represents an architectural concept related to the E Group.

The tradition of the MFC pattern with an E-Group assemblage on the southern Gulf Coast and in Chiapas essentially ceased at the end of the Middle Preclassic period with the collapse of La Venta and related centers. In the central and eastern Maya lowlands, in contrast, a large number of E Groups were built during the Late and Terminal Preclassic periods [17,64,65], leading to the development of Uaxactun-type E Groups in the Terminal Preclassic and Early Classic periods. The E-Group assemblage of Tikal took the form of the Uaxactun type during the Terminal Preclassic Cauac phase, which we would date to 75 BC-AD 100, whereas at Uaxactun and Caracol this form developed during the Early Classic [17,45]. At Cahal Pech and other sites in the Belize River valley, what Awe et al. call Eastern Triadic Structures (three buildings constructed in a row with or without a paring western structure) were built as early as the late Middle Preclassic period. Structures in some of these complexes, however, were originally unconnected, built and renovated at different timings, and it is not clear whether they represent an architectural concept directly tied to the E Group [66]. The Uaxactun type of E Group was never adopted in the Ceibal region, possibly because of the declining popularity of E Groups in the area after the Late Preclassic period, which may have been related to the collapse of MFC centers in Chiapas and on the Gulf Coast.

During the Late and Terminal Preclassic periods, the Triadic Group with three structures set on a pyramidal base became another characteristic format for ceremonial complexes in central Maya lowlands [16,67]. This configuration is found in Group D of the Ceibal center, dating to the Terminal Preclassic period and later [26,42]. In other parts of the Ceibal region, however, this format is nearly absent. Potential examples include the Palacio Group (Structures 3H-1 and 3H-5) and Structure 10G-1 of Group B, but their triadic arrangements are ambiguous. Although excavations did not reach the core part of the main pyramid of the Palacio Group, the earliest layer of the plaza dated to the Junco 1 phase (AD 175–300). The limited use of the triadic group in the Ceibal region parallels the unpopularity of the Uaxactun-type E Group, both indicating the limited sharing of architectural formats with the central lowlands, despite similarities in ceramic styles between those regions during the Late and Terminal Preclassic periods.

Many residential groups during the Late Preclassic period appear to have consisted of supporting platforms. In Group A, the extensive A-24 Platform located southwest of the E-Group assemblage was one of the earliest buildings, along with the E-Group structures, constructed at the beginning of the early Middle Preclassic Real 1 phase, although it is not clear whether it served as a residential complex. The construction of another platform, the East Court, located northeast of the E Group, began during the Real 3 phase, and it was most likely used as a residential group of emergent elites. Outside Group A, excavations by Jessica MacLellan have shown that the Karinel Group (Unit 47) was occupied during the Real 2 phase and the construction of its supporting platform began during the late Middle Preclassic period [33]. At the Jul Group (Unit 54), the naturally high area was used at least by the end of the Middle Preclassic, and the first construction fill of the platform was placed at the beginning of the Late Preclassic. A platform in the Amoch Group (Unit 4E-14) appears to have been built initially during the Late Preclassic Cantutse phase, Platform 97 during the Cantutse 2 phase, and Platform C-32 during the Cantutse 1 or 2 phase (Fig 3). In addition, excavations by the Harvard Project indicate that Platforms 10, 25, and 9F-1 were constructed during the late Middle Preclassic, Late Preclassic, and Terminal Preclassic periods [15]. All of these excavated platforms continued to be occupied during the Late Preclassic, although the use of some locations declined during the Terminal Preclassic.

Some Preclassic residential groups, particularly ones during the early Middle Preclassic period, did not include supporting platforms, but consisted of individual structures built directly on bedrock or thin floor layers. If those groups were not built over during later periods, they are difficult to detect in LiDAR analysis or surface survey.

#### Classic-period architecture

Excavation results at Ceibal indicate that the center experienced a significant population decline at the end of the Early Classic Junco 1 phase around AD 300, when some peripheral temple complexes, including those in the Muknal, Pek, and Amoch Groups, were ritually buried with stony black earth and abandoned [28,68]. As Ceibal regained its population during the Late Classic period, various pyramidal complexes, including Groups A and D, Anonal, and Caobal, regained construction activity. However, those that were ritually buried during the Junco phase were never formally rebuilt, and it is not clear whether they functioned as ritual foci during the Classic period. Some pyramidal complexes, including those in the Palacio and Jul Groups, may have been rebuilt during the Classic period.

Abundant excavation data show that a significant number of rectangular structures of low to medium heights were built during the Classic period, particularly the Late and Terminal Classic. This is a common pattern found throughout the Maya lowlands [69,70]. These buildings were predominantly domiciles and associated domestic structures [15]. Many supporting platforms of the Preclassic period were reoccupied during the Classic period, and structures visible on these platforms, often surrounding patios, commonly date to this period.

### Excavation and surface collection at sites identified in LiDAR

During the 2016 and 2017 seasons, we conducted test excavations at two of the Cenote-type E-Group assemblages that were identified in the LiDAR data: La Felicidad (Unit AD10-4) and El Edén (Unit AB14-25) (Fig 2). We placed test units of 2 x 1 m or 1 x 1 m in the E-Group plazas and in areas around them to examine the occupation history of the sites. At El Edén, we also cleaned a looters’ trench for the length of roughly 4 m on the eastern side of the east building of its E-Group assemblage (Structure AB14-79). Although we did not reach the core parts of the pyramids, the excavations in the plazas penetrated all construction layers down to bedrock.

The earliest plaza construction at La Felicidad dates to the early Middle Preclassic Real 3 phase (775–700 BC). The plaza was remodeled various times during the late Middle Preclassic, Late Preclassic, and Terminal Preclassic periods, and then covered by thin floor fills during the Late and Terminal Classic. At El Edén, the lowest plaza fill dates to the late Middle Preclassic period (700–350 BC). Because the number of ceramics found in this excavation was small, we could not date the earliest construction to a specific facet. The plaza underwent multiple additions of floor layers during the Late Preclassic and Late Classic periods, but we did not find diagnostic Terminal Classic ceramics at this site. In the clearing of the looters’ pit at El Edén, we identified a substantial construction (Structure AB14-79 Sub-1) dating to the Late Preclassic period underneath the eroded final construction layer of the Late Classic period. Below Structure Sub-1 was a sequence of thin floors dating to the late Middle Preclassic period, but we did not reach bedrock. These floors were probably associated with a late Middle Preclassic-version of the pyramid located in the unexcavated core part.

These results align with findings from the Ceibal center. It is probable that the E-Group assemblages of La Felicidad and El Edén began during the late part of the early Middle Preclassic or during the late Middle Preclassic period. It is not clear, however, whether the E-Group assemblages of these sites were originally of the La Venta type or whether they were built as the Cenote type from their inception.

Along with the ground-truthing of the LiDAR data during the 2016 season, we collected artifacts from the surface of visited sites (Fig 2). Our surface collection focused on looters’ pits and other disturbances that are commonly found outside the protected Ceibal Park. The use of surface collection is generally limited in the southern Maya lowlands because artifacts are almost absent on the surface in the undisturbed areas of rainforest. Even in areas with other vegetation types, the quantity of artifacts visible on the surface is small because plowing is uncommon in the region, and open grounds without vegetation cover are limited. In addition, at many lowland Maya sites where early occupation layers are buried under later constructions, surface materials may not reflect early occupation well. In excavations in the Ceibal center, artifacts recovered from the surface level included no sherds from early periods or only a minimal quantity of them, despite the presence of substantial early constructions revealed in deep excavations (Table 2). Thus, Maya archaeologists have relied mainly on test-pitting to obtain chronological information on settlement patterns [15,69,71–73]. The settlement data of the Ceibal region need to be examined with a more extensive test-pitting program in the future. Nonetheless, it becomes increasingly challenging to obtain representative samples with test excavations as LiDAR-surveyed areas rapidly expand. As an expedient substitute for test excavation for the purpose of an initial evaluation, we implemented the surface collection program focused on looters’ pits. We collected materials found on the surface when possible, but a substantial portion of collected artifacts came from disturbed areas.

**Table 2:** Ceramics excavated from the surface level (above the final floor) in Ceibal Group A.

Suboperation	Early Middle Preclassic	Late Middle Preclassic	Late and Terminal Preclassic	Early Classic	Late Classic	Terminal Classic	Post-classic	Non-diagnostic	Total
CB200B^a^			25		3	1		150	179
CB201A	2	1	51		43	17		572	686
CB201B			24		6	4		217	251
CB201F			1		8			39	48
CB202A			86		10	2		186	284
CB203A								7	7
CB203B			61		10	1		258	330
CB203C								1	1

^a^They represent suboperations that had excavation areas larger than 12 m^2^ and revealed occupation layers of the early and late Middle Preclassic periods.

We collected identifiable ceramic materials at 178 units of structures and platforms. Many sherds were classified into the broad temporal divisions of the Preclassic, Early Classic, and Late-Terminal Classic. The dating of Preclassic sherds remained coarse. Although the Harvard researchers classified many sherds from windfall collections (surface collections from locations disturbed by tree falls) and other mixed contexts into specific Preclassic phases [26,68], we preferred a more conservative approach because our ceramic studies suggested substantial overlaps in ceramics styles across these phases. In most cases, we classified sherds into groups of phases, such as Preclassic general (early Middle to Terminal Preclassic), Escoba-Cantutse-Xate (Late Middle, Late, and Terminal Preclassic), Cantutse-Xate (Late and Terminal Preclassic), and Tepejilote-Bayal (Late and Terminal Classic) (Tables 3 and 4).

**Table 3:** Surface collection ceramic frequencies and percentages by periods and phases.

		Preclassic					Classic						Postclassic	Total IDed	Eroded
Unit type	Freq. of Units^a^	General^b^	late Middle-Terminal^c^	Late-Terminal^c^	Terminal	Subtotal	Genera^b^	Early	Late-Terminal^c^	Late	Terminal	Subtotal			
E Group	4	0.0%	41.2%	14.7%	2.9%	58.8%	20.6%	0.0%	8.8%	2.9%	8.8%	41.2%	0.0%	34	8
Possible E Group	2	0.0%	0.0%	0.0%	0.0%	0.0%	89.5%	5.3%	5.3%	0.0%	0.0%	100.0%	0.0%	19	0
Eastern winged pyramid	2	5.6%	5.6%	5.6%	0.0%	16.7%	5.6%	0.0%	16.7%	55.6%	5.6%	83.3%	0.0%	18	1
Pyramid & platform	2	0.0%	21.3%	27.7%	17.0%	66.0%	19.1%	0.0%	2.1%	4.3%	8.5%	34.0%	0.0%	47	9
Pyramid without platform	3	7.0%	23.3%	34.9%	7.0%	72.1%	0.0%	0.0%	14.0%	0.0%	14.0%	27.9%	0.0%	43	13
Supporting platform	56	7.6%	12.1%	4.5%	8.4%	32.6%	10.5%	0.2%	26.0%	27.8%	2.9%	67.4%	0.0%	619	165
No supporting platform	109	0.0%	8.1%	1.4%	3.0%	12.5%	14.7%	0.7%	44.5%	24.6%	2.5%	87.0%	0.5%	1062	275
Total	178	2.8%	10.6%	4.2%	5.2%	22.8%	13.8%	0.5%	35.2%	24.2%	3.2%	76.9%	0.3%	1842	471

^a^Units without identifiable sherds are not counted.

^b^Percentage of sherds dated to the Preclassic or Classic period and not to finer phases. Percentages are calculated in relation to the total number of identified shards (Total IDed).

^c^Percentage of sherds dated to these periods and not to a finer period or phase.

**Table 4:** Frequencies of units where Preclassic or Late-Terminal Classic sherds were present.

	Unit frequencies^a^		
Unit type	Preclassic	Late-Terminal Classic	Total
E Group	2	1	2
Possible E Group	0	1	1
E Winged	1	1	1
Pyramid & platform	2	2	2
Pyramid without platform	2	1	2
Supporting platform	14	17	17
No supporting platform	18	33	33
Total	39	56	58

^a^Units with ten or more identified sherds are counted.

We did not find any ceramics that can be confidently dated to the early Middle Preclassic period. This pattern is partly because of the difficulty in identifying ceramics of this period in this mixed and eroded collection. As the early Middle Preclassic materials recovered in excavations at Caobal and La Felicidad indicate, there were settlements dating to this period in some areas outside the Ceibal center. Still, the population, or the ceramic-using population, was likely low during this period.

The high ratio of Preclassic ceramics found at units with E-Group assemblages confirms our assumption that these sites were originally constructed during the Preclassic period and reoccupied during the Classic period. The presence of one E Group without Classic sherds in Table 4 may be because surface collection focused on looters’ trenches cutting into Preclassic fills. All the E-Group assemblages found outside the Ceibal center were of the Cenote type (Fig 8). Although our data do not provide a fine chronology of their construction, the high percentage of sherds belonging to the Middle-Terminal Preclassic and the low ratio of the Late-Terminal Preclassic sherds are congruent with the observation that Cenote-type E Groups began to be built during the late Middle Preclassic period. In contrast, at the units classified as “possible E Groups,” we did not find any Preclassic ceramics. This pattern may be due to a sampling error resulting from the small number of units of this type that were visited and the small quantity of sherds collected there. Nonetheless, these groups do not show regular and symmetrical arrangements of architecture common in the confirmed E-Group assemblages. Given these results, we tentatively interpret these groups as configurations different than E Groups.

At eastern winged pyramids without pairing western pyramids, the percentage of Preclassic ceramics was surprisingly low. This pattern, which diverged from our expectation, may be due to sampling errors. We collected surface artifacts at only two units of this type, and the number of collected sherds is small. One was the minor center of Caobal (Unit AD12-18). As described above, intensive excavations by Munson showed that the occupation of the site began in the early Middle Preclassic Real 2 phase, and the winged pyramid was first built during the late Middle Preclassic Escoba 2 phase. Nonetheless, the quantities of ceramics in Preclassic construction fills were generally small, and the center had substantial Late and Terminal Classic activities. These conditions possibly contributed to the small ratio of Preclassic materials among surface-collected materials. Another unit was AG15-8, located 2.5 km to the southwest of Group A. LiDAR shows that the winged pyramid of this unit is surrounded by large rectangular structures, which most likely served as an elite residential complex of the Classic period. Again, this occupation history may have resulted in a high ratio of Classic materials. Thus, these results do not necessarily deny the assumption that most winged pyramids were originally built during the late Middle Preclassic or Late Preclassic periods.

Other pyramidal complexes, including those associated with supporting platforms (pyramid & platform) and those without them (pyramid without platform), show high percentages of Preclassic ceramics. This pattern supports the view that a significant portion of pyramidal-temple complexes were built during the Preclassic period. More specifically, their ratios of ceramics dating to the Late or Terminal Preclassic and to the Terminal Preclassic are higher than those of the E-Group assemblages, which is consistent with the assumption that temple pyramids that were not parts of E Groups or winged pyramids were mostly built during the Late or Terminal Preclassic period. The category of late Middle-Terminal Preclassic ceramics means that the materials can be dated only to this broad range, and the presence of these ceramics does not necessarily indicate that their construction began during the late Middle Preclassic. The presence of Classic-period sherds suggests that most of these groups were reoccupied during the Classic period.

As expected, units with supporting platforms had higher ratios of Preclassic ceramics than those without platforms (No supporting platform). Nonetheless, their percentage of Preclassic materials is lower than those at the E Groups or other pyramidal complexes. This pattern is probably due to the facts that many of those platforms were reoccupied during the Late and Terminal Classic periods and that at those sites looter’s pits were commonly dug into Classic-period structures. In addition, a substantial portion of units without platforms had Preclassic sherds (Table 4). This pattern may be because these units had Preclassic platforms that were not identified in LiDAR analysis. When Classic-period patio groups were built over low supporting platforms, it is difficult to tell without excavation whether they had supporting platforms.

The results of the test excavations and surface collection at sites identified in the LiDAR data generally accord with the architectural chronology suggested by the intensive excavations carried out in the Ceibal center. In terms of E-Group assemblages, the La Venta type and the associated MFC pattern have not been found outside Group A, although the possibility of the former cannot be ruled out. All E-Group assemblages outside Group A were of the Cenote type in their final forms, which may have been constructed initially during the late Middle or Late Preclassic period. The surface collection data generally support the assumption that supporting platforms were constructed during the Preclassic period. Most pyramidal complexes and supporting platforms were reoccupied during the Classic period.

## Settlement dynamics

The settlement dynamics of the Ceibal region needs to be examined with more extensive excavation data in the future. Here we present an initial synthesis of settlement dynamics in the Ceibal region by combining the results of our investigations associated with the LiDAR survey and those of the previous excavations. Given the coarse chronological resolution of surface-collection materials, our analysis of residential structures and population distribution focuses on: 1) the Late Preclassic period when the population of the Ceibal center reached its first peak; and 2) the Late-Terminal Classic period which witnessed the second and largest population peak. A more detailed architectural chronology for the Preclassic period was developed for ceremonial complexes based primarily on the intensive excavations of Group A and on smaller excavations in other parts of the Ceibal center. Out of the ten E-Group complexes in the region, which make the core part of the architectural chronology, four (Group A, Anonal, La Felicidad, and El Edén) have been excavated and two others (La Nueva Felicidad and El Rodeo) have been targets of surface collection. Out of the seven east winged-pyramid complexes, two (the Pek Group and Caobal) have been excavated, and another (Unit AG15-8) has been visited for surface collection. The data on other pyramidal complexes are represented by excavations at Group D (Pyramidal Structures D-31, D-32, D-33, and D-41), the Palacio Group, the Muknal Group, the Amoch Group, and Structure 1H-10 in the Ceibal center, at Structure 8C-3 outside of the center, and surface collection at seven complexes identified in the LiDAR data. Although this chronology needs to be tested with more excavations, these data provide reasonable support for the general applicability of this architectural chronology to the entire study area.

### Preclassic period

**Fig 9:**
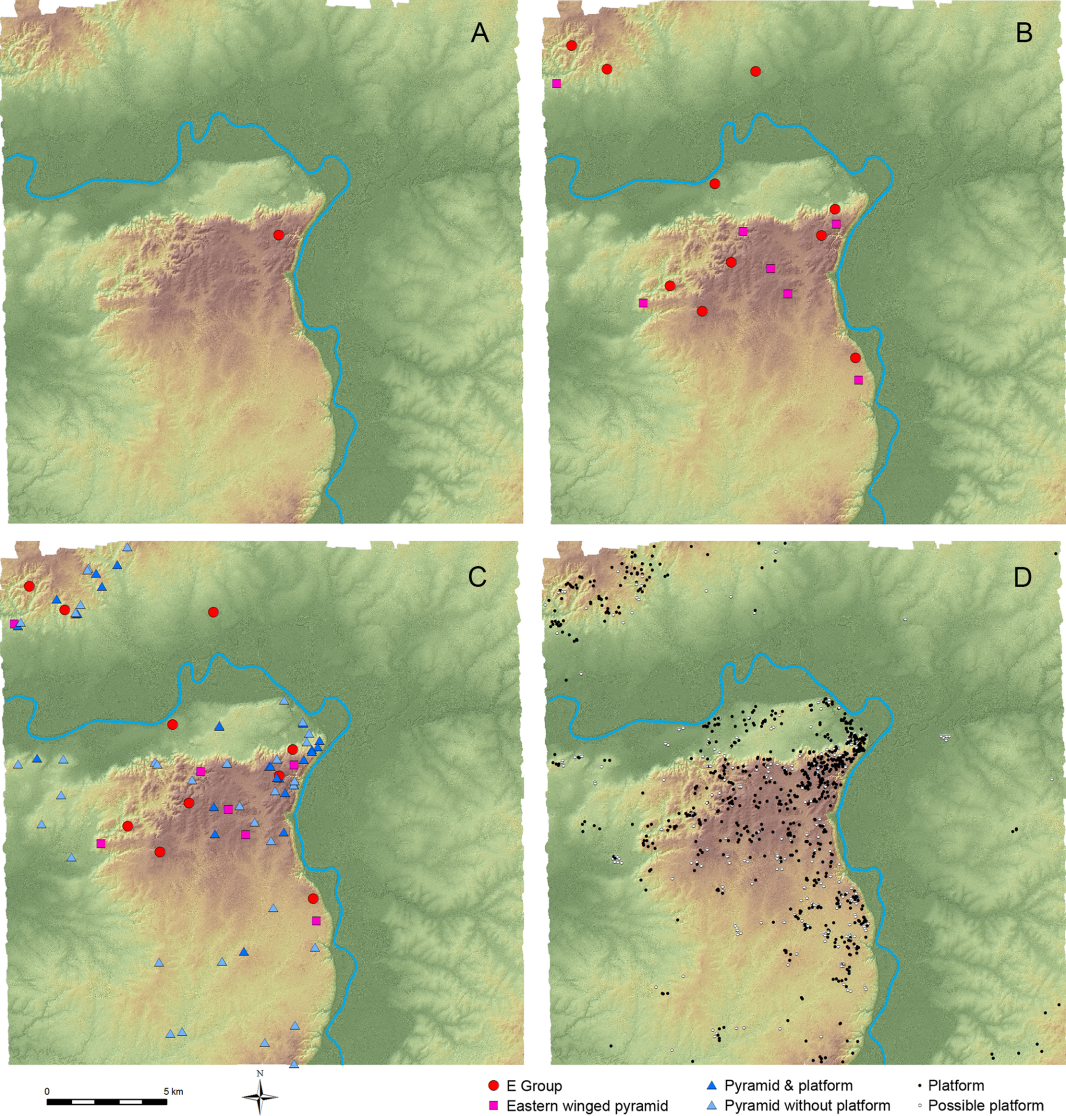
Estimated distribution of ceremonial complexes and supporting platforms during the Preclassic period. (A) MFC pattern with a La Venta-type E Group from the early Middle Preclassic Real 1 to late Middle Preclassic Escoba 1 phase (1000–600 BC). (B) Cenote-type E Groups and eastern winged pyramids, possibly dating to the late Middle Preclassic Escoba 2 to Late Preclassic Cantutse 1 phase (600–300 BC). (C) Pyramidal complexes, possibly dating to the Late Preclassic Cantutse 1 to Early Classic Junco 1 phase (350 BC-AD 300). Units originally classified as “possible E Groups” are included in “pyramid without platform.” (D) Supporting platforms (probable residential complexes during the Preclassic period).

For the early Middle Preclassic Real 1 phase (1000–850 BC), the La Venta-type E Group at Group A is the only confirmed ceremonial complex in this region (Fig 9A). Even in the intensively studied Ceibal center, we did not find any other pyramids, platforms, or residential structures dating to this phase outside of Group A. We suspect that the Ceibal region during this phase was mostly devoid of durable constructions besides the Group A complex. This does not necessarily mean that areas outside Group A were unoccupied. The substantial construction of the Group A complex would have required a considerable number of builders. We have suggested elsewhere that a significant part of the population in the Ceibal region was still practicing a mixed subsistence economy, combining fishing, hunting, gathering, and maize cultivation [74]. They probably lived in ephemeral buildings, such as those with posts in ground, and retained considerable mobility, moving either seasonably between cultivation plots and fishing-hunting-gathering areas or every few to several years according to swidden cultivation cycles.

Intensive excavations at Ceibal indicate that during the early Middle Preclassic Real 2 and 3 phases (850–700 BC), the population gradually increased, or people were becoming less mobile. We suspect that this was a region-wide pattern. Traces of post-in-ground structures, ceramic deposits over scraped bedrock, and ceramics of the period mixed in later construction fills have been found at the Karinel Group and other units around Group A, at Caobal, and at La Felicidad. With the possible exception of Structure 79 located south of Group C, none of these areas yielded evidence of substantial construction during this period [15]. It is difficult to identify these subtle traces of occupation without intensive excavations, and the overall population distribution of the early Middle Preclassic is not known.

During the late Middle Preclassic Escoba 1 and 2 phases (700–450 BC), small structures with basal platforms were built at the Karinel Group and Caobal, suggesting that some part of the population was becoming more sedentary. From the Escoba 2 phase to the Late Preclassic Cantutse 1 phase (600–300 BC), the Cenote-type E Groups and eastern winged pyramids that did not form E Groups probably began to be built, as suggested by the excavation results from Group A, the Pek Group, Anonal, and Caobal (Fig 9B). Nonetheless, we do not deny the possibility that some of these E Groups and the winged pyramids were initially built during the Escoba 1 phase or earlier.

From the Late Preclassic Cantutse 1 phase (350–300 BC) through the Early Classic Junco 1 phase (AD 175–300), pyramidal complexes that did not form E Groups or did not have eastern winged pyramids appear to have been built. Fig 9C is based on the assumption that these non-E-Group pyramids, including those with or without supporting platforms, as well as the complexes originally classified as “possible E Groups,” belong to these periods and that the E Groups continued to be in use. However, some of these complexes may have been built earlier, and others may not have been built until later periods. Thus, in reality the number of pyramidal complexes belonging to the Cantutse 1-Junco 1 time span may be somewhat smaller than the pattern in Fig 9C, but this image likely reflects a general trend.

While these complexes with pyramids constituted foci of public ceremony, supporting platforms indicate the distribution of residences over the landscape. These platforms, however, were built throughout the Preclassic period, and we cannot determine the dates of their initial constructions without penetrating excavations. In addition, some parts of the population, particularly those in the Middle Preclassic period, possibly lived in ephemeral post-in-ground structures, which cannot be detected by LiDAR. Thus, it is difficult to estimate the population distribution of the Middle Preclassic period. The results of investigations by the Harvard Project and the CPAP suggest that the population in the Ceibal center and its immediate vicinities increased during the Late Preclassic Cantutse phase and then declined to some degree during the Terminal Preclassic Xate phase [15,36]. Although we do not have sufficient data to fully evaluate whether the same demographic trend applies to other parts of the Ceibal region, the results of excavations at La Felicidad and El Edén are suggestive of its applicability. In addition, excavations of similar supporting platforms at Aguateca showed that they were built during the Late Preclassic period [75]. If we assume that the demographic trends of the Ceibal center and Aguateca are applicable to the entire Ceibal region, the distribution of supporting platforms shown in Fig 9D may reflect the population pattern during the Late Preclassic Cantutse phase.

Table 5 summarizes the results of ground-truthing supporting platforms. Our ground-truthing data suggest that, unlike small structures, supporting platforms with extensive horizontal dimensions can be identified reasonably well in LiDAR analysis under different vegetation types. Misidentifications of supporting platforms resulted mainly from the difficulty in interpreting features affected by later constructions and erosion. Out of 13 field-identified platforms (ones that were not identified in LiDAR analysis), 12 were low platforms on which later buildings were added. Out of five discarded features, two were eroded structures (one was identified as a “possible platform”), two modern constructions, and one a naturally elevated area. To calculate the population of the Ceibal region, we followed the formula used by Tourtellot [76]. He assumed that 90% of the platforms were contemporaneously occupied and that an average of 11.9 persons lived on a platform. The excavation of the East Court and the Karinel Group by the CPAP suggested that some platforms supported multiple residences surrounding a patio, which help support this assumption. Nonetheless, we should note that these population estimates may contain substantial errors.

**Table 5:** Ground-truthing and estimates of supporting platforms.

Zone	Classification	LiDAR analysis	Ground-truthing				Accuracy		Platform estimate		Population estimate	
			Target verified	Positive verification	Discarded	Field IDed^a^	Commission error^b^	Omission error^c^	Frequency^d^	Density (/km^2^)	Frequency^e^	Density (/km^2^)
Ceibal center	Platform	196	0						247.2	45.70	2,648	489.4
	Possible platform	30	0									
	Non-domestic	10	0						10.0	1.85		
Ceibal horst^f^	Platform	405	66	62	4	8	6.1%	11.4%	627.5	4.91	6,720	52.6
	Possible platform	187	13	12	1		7.7%					
	Non-domestic	17	2	2					17.0	0.13		
Subín horst	Platform	75	10	10		5	0.0%	33.3%	97.9	3.62	1,048	38.8
	Possible platform	15	2	2			0.0%					
	Non-domestic	8	2	2					8.0	0.30		
Other	Platform	13	0						34.1	0.11	366	1.2
	Possible platform	21	0									
	Non-domestic	0	0						0.0	0.00		
Entire area	Platform	689	76	72	4	13	5.3%	15.3%	1,006.7	2.14	10,782	22.9
	Possible platform	253	15	14	1		6.7%					
	Non-domestic	35	4	4					35.0	0.07		
Total		977	95	90	5	13			1,041.7	2.22		

^a^Not identified as platforms in LiDAR and identified in ground-truthing.

^b^Falsely identified as platforms in LiDAR analysis = Discarded/Target verified.

^c^Falsely not identified as platforms in LiDAR analysis = Field IDed/(Positive verification + Field IDed).

^d^Estimated frequencies of features in each zone. For the categories of “platform” and “possible platform,” we first calculated the estimated frequency in each category, which is given as: *L*–*C*_*e*_+*O*_*e*_ = *L*-*LC*_*g*_+*LO*_*g*_(1-*C*_*g*_)/(1-*O*_*g*_) = *L*[1-*C*_*g*_+*O*_*g*_(1-*C*_*g*_)/(1-*O*_*g*_)], where *L* is the frequency of platforms or possible platforms identified in LiDAR analysis; *C*_*e*_ is the estimated commission (the estimated frequency of features falsely identified as platforms in LiDAR analysis); *O*_*e*_ is the estimated omission (the estimated frequency of features falsely not identified as platforms in LiDAR analysis); *C*_*g*_ is the commission error rate calculated with the ground-truthing results; and *O*_*g*_ is the omission error rate calculated with the ground-truthing results. *C*_*g*_ and *O*_*g*_ for the entire area are used for individual zones. We then added the calculated numbers for “platforms” and “possible platforms” to obtain the total estimated frequency of “platforms.” “Non-domestic” includes generally large platforms associated with pyramids and other public buildings, and we assume that all of them were identified in LiDAR analysis.

^e^Population is calculated as: estimated platform frequency (excluding non-domestic ones) x 0.9 (rate of contemporaneously occupied platforms) x 11.9 (persons/platform).

^f^Ceibal horst not including the Ceibal center.

**Fig 10:**
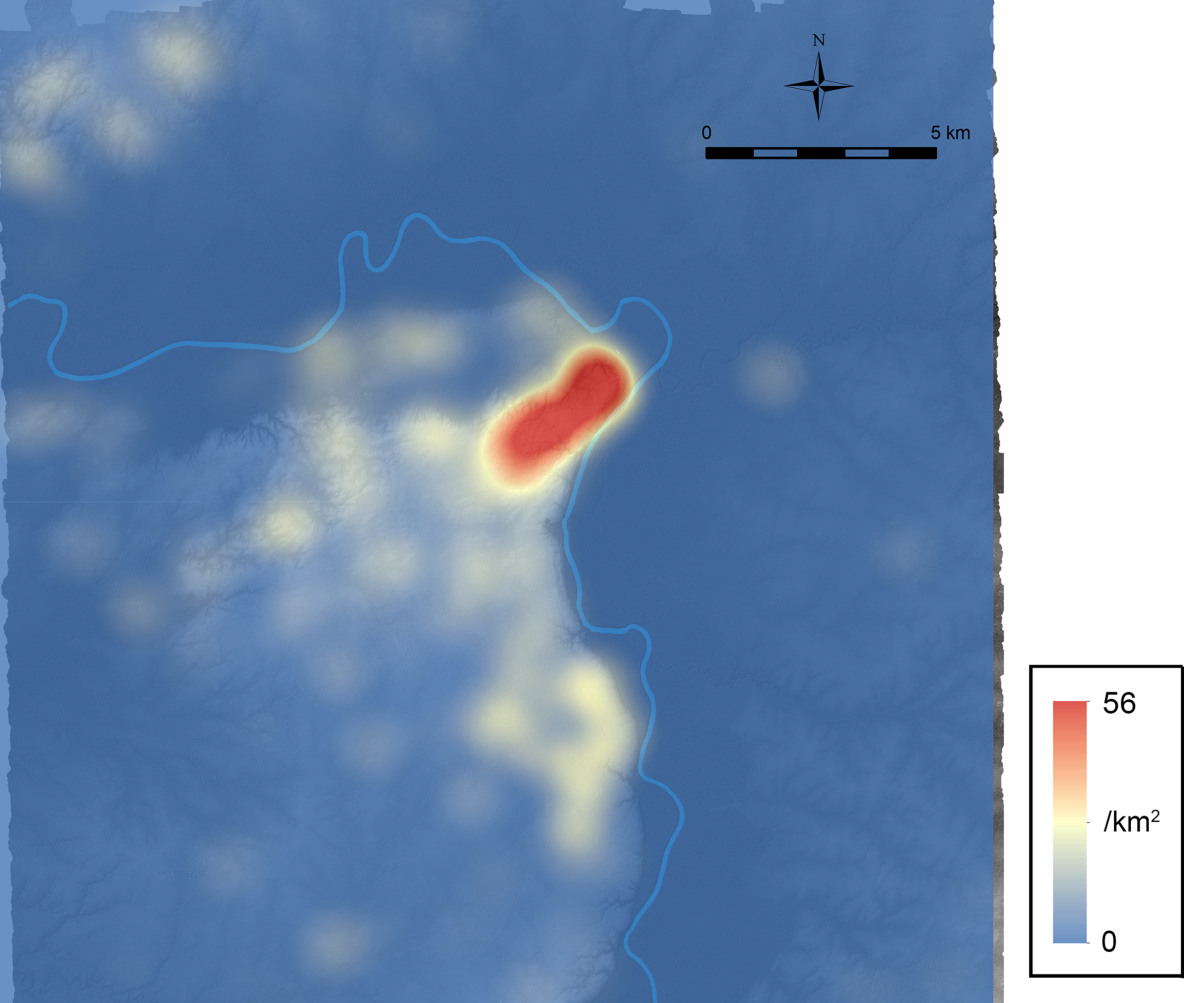
Platform density without non-domestic platforms. It likely approximates the population distribution of the Late Preclassic period (350–75 BC) if our assumption that the Preclassic population in both in the Ceibal center and in the peripheries reached its peak during this period is correct.

Fig 10 shows the platform density without non-domestic platforms, which possibly reflects the distribution of population during the Late Preclassic period. To generate this figure, we used the kernel density tool of ArcMap with the default search distance. The basis of density calculation was a feature table of platforms identified in LiDAR analysis, excluding non-domestic ones, in which the population field contained the variable [1-*C*_*g*_+*O*_*g*_(1-*C*_*g*_)/(1-*O*_*g*_)] used for the calculation of estimated frequencies in Table 5 (1.118 for “platforms” and 0.933 for “possible platforms”). The values in the population field adjust the results of LiDAR analysis to generate the density distribution of the estimated platform counts based on ground-truthing data. Fig 10 and Table 5 show a high concentration of population in the Ceibal center. This pattern correlates with the similar clustering of pyramidal temples shown in Fig 7C. Moderate concentrations of population are found around El Edén, Anonal, Iberia, and in the Subín horst. If our assumption that the Preclassic peripheral population reached its peak during the Late Preclassic period is not correct, it would mean that the population concentration in the Ceibal center during the Late Preclassic period was even stronger than this figure suggests.

### Classic period

Excavations by the Harvard Project and by the CPAP show that a substantial part of the Ceibal center was abandoned at the end of the Early Classic Junco 1 phase (AD 175–300), and the population of the Ceibal center during the rest of the Early Classic period remained extremely low [36]. Although Harvard researchers recovered Junco ceramics at a considerable number of locations [15], we suspect that a substantial part of these ceramics were of the Junco 1 phase. The paucity of the Early Classic ceramics in the surface collection and the excavations outside the Ceibal center suggests that a comparable population decline occurred throughout the Ceibal region. Nonetheless, outside the Ceibal center we have not found evidence of temples buried in black soils comparable to the cases of some central pyramids. During the Late Classic Tepejilote phase, the Ceibal center regained a high population level, and similar demographic trends in other zones are suggested by our LiDAR data and the surface collection. However, the data based on LiDAR and surface collection are not adequate for more precise dating within the Late-Terminal Classic period. For the following analysis, we simply assume that the distribution of Classic-period buildings approximates the settlement pattern at the population peak during the Late or Terminal Classic period.

During the Late and Terminal Classic periods, social stratification centered around the ruler was well established, and there existed substantial differences in the size and elaborateness of residential buildings in addition to those of temples and shrines. To examine social organization of these periods, we ranked structure units by examining the sizes of pyramids, residential structures, and other associated constructions (Table 6). In developing this ranking, we assumed that all platforms and pyramids of the Preclassic period were reoccupied during the Classic period. We should note that a significant portion of the construction masses of some pyramids and platforms may have been built during the Preclassic period and that those pyramids buried at the end of the Early Classic Junco 1 phase may not have been reused as temples in later periods. Nonetheless, an important criterion for ranking is the size of palace-type buildings (probable elite administrative-residential buildings) and other residential structures. Thus, the distribution of these ranked units should reasonably reflect political organization in the Ceibal region during the Late and Terminal Classic periods. In addition, not to skew spatial distribution, this ranking does not include units consisting only of features originally classified as “possible structures” or “possible supporting platforms.” Nor does it include those added after the ground-truthing. Most of those units or possible units belong to the smallest end of Rank 1, and thus their exclusion does not affect the distribution of larger units.

**Table 6:** Ranking of structure units.

Rank	Frequency	Definition
1	2,997	Small residential unit
2	304	Medium-size unit (width greater than 30 m or a mound higher than 1.5 m)
3	75	Large unit (width greater than 50 m or a mound higher than 3 m)
4	32	Minor center: palace-type buildings or pyramid taller than 5 m.
5	6	Medium-size center: substantial palace-type buildings or pyramid taller than 10 m. (Unit A-3 in Group A, Units D-32 and D-34 in Group D, Amoch Group, Palacio Group, and Anonal)
6	2	Major center: pyramid taller than 20 m (Units A-10 and A-24 in Group A)

**Fig 11:**
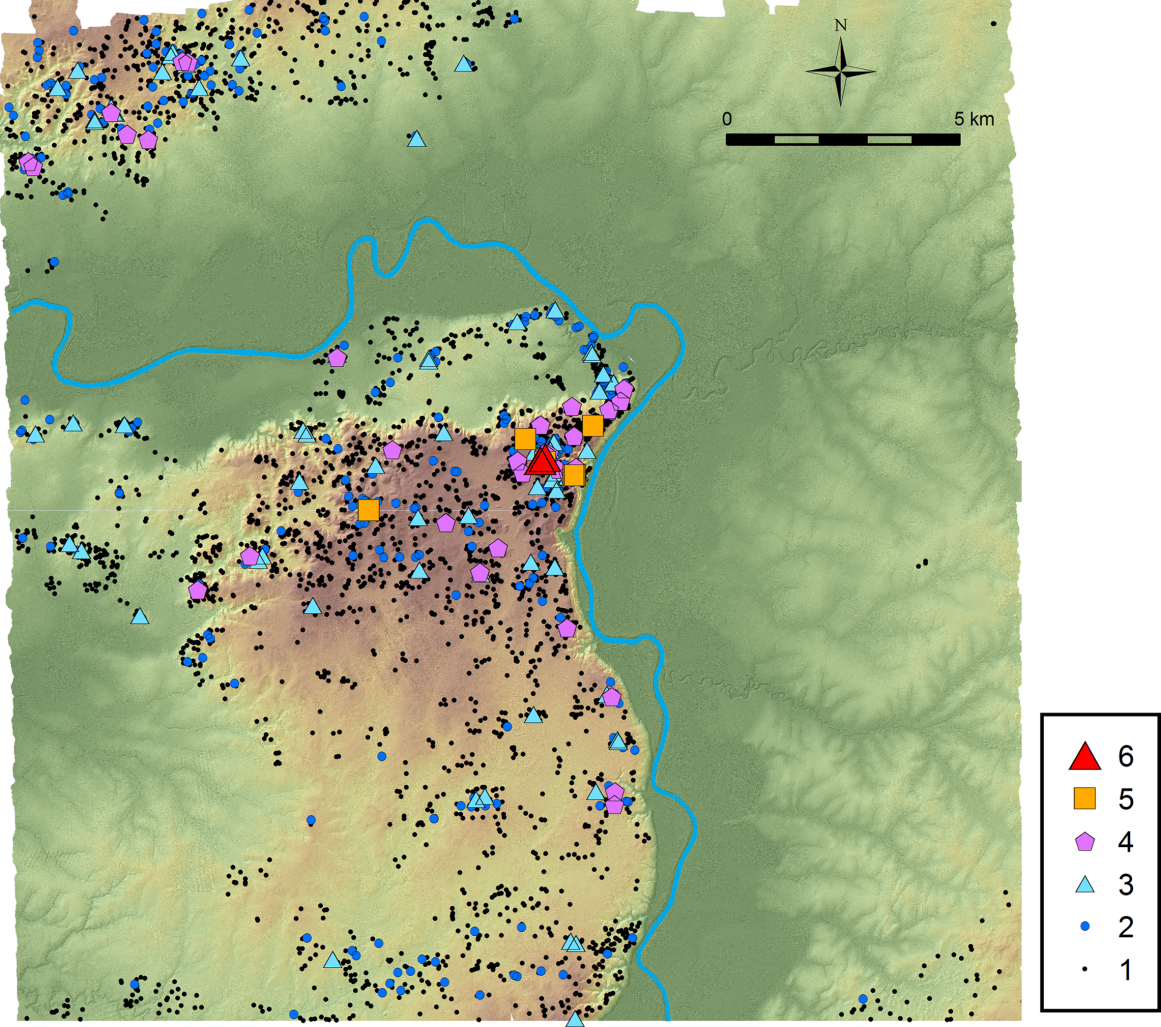
Distribution of units by ranking. It probably reflects the political organization during the Late and Terminal Classic periods.

**Fig 12:**
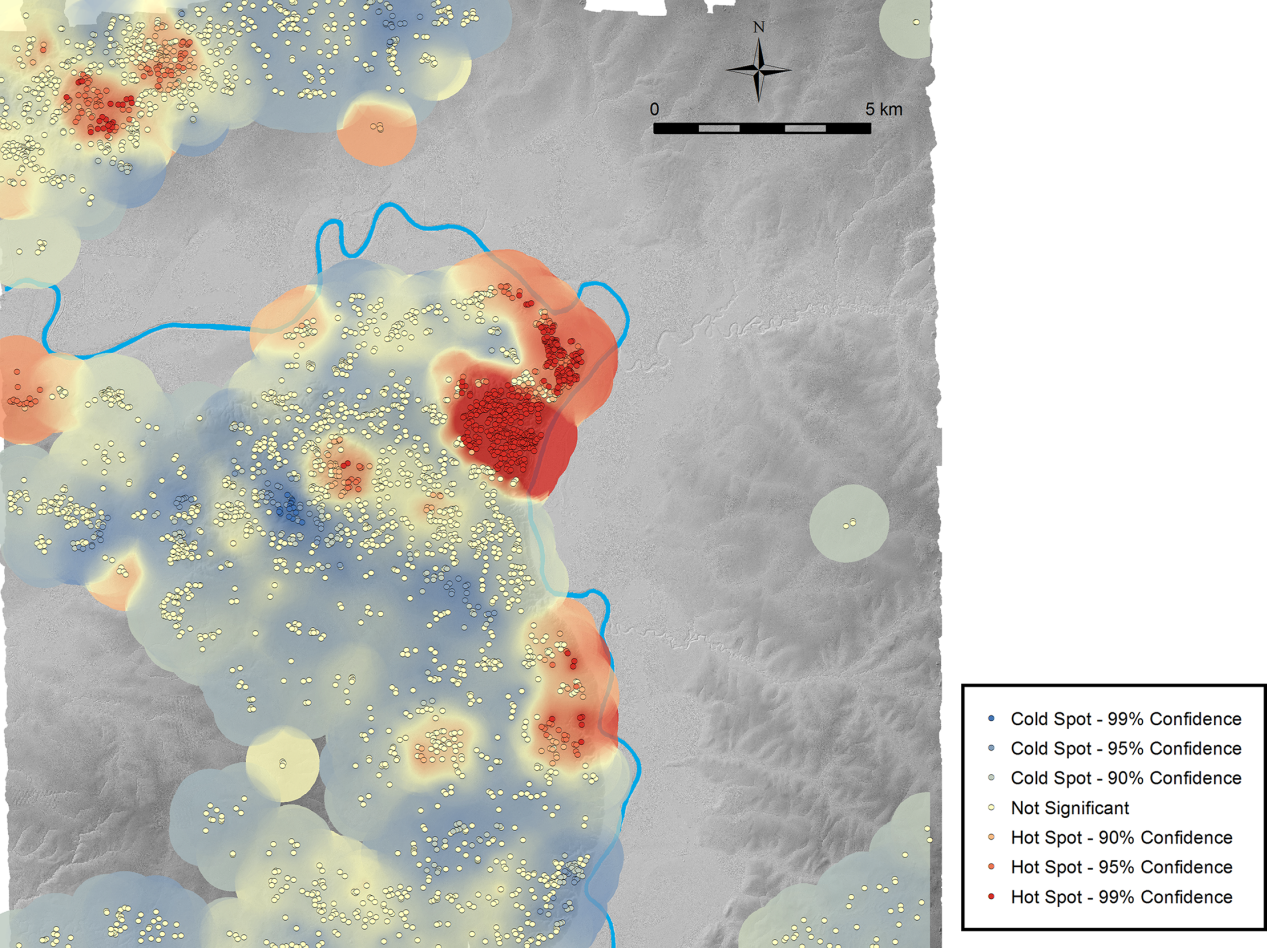
The result of hot spot analysis based on the unit ranking. Cold spots represent statistically-significant clustering of low-rank units, whereas hot spots show statistically-significant clustering of high-rank units.

Fig 11 shows a concentration of high-rank units in the Ceibal center. The result of spatial statistics confirms this intuitive understanding (Fig 12). This analysis used ArcMap’s Hot Spot Analysis tool, or the Getis-Ord Gi* statistics, by applying a fixed distance band of 850 m obtained as a peak in z-scores calculated with ArcMap’s Incremental Spatial Autocorrelation tool, or Moran’s I statistics. The highest clustering of high-rank units exists in the Ceibal epicenter and its immediate surroundings, and the northern part of the Ceibal center also exhibits a high concentration. Other clustering of high-rank units are found around the satellite center of Anonal, the site of Iberia, and in the Subín horst around the sites of El Rodeo and La Jutera. At Anonal, a Terminal Classic panel referring to the Ceibal ruler was found, indicating the political importance of this location [77].

To examine the population level, density, and distribution during the Late and Terminal Classic periods more specifically, we need to focus on residential structures. The results of the previous excavations and the surface collection suggest that a substantial portion of features identified as structures both in the Ceibal center and in the peripheral zones date to these periods. Many structures are small in terms of both horizontal and vertical dimensions, and their detection in the LiDAR data was affected by vegetation cover. The analysis of LiDAR effectiveness in different vegetation types and the results of ground-truthing are discussed in detail in a previous publication [22]. In the present study, we combined some vegetation types so that each area has a sufficient number of ground-truthed samples. In addition, we separately analyzed probable non-domestic structures, such as temples, shrines, ballcourts, and public buildings surrounding large plazas. Most palace-type buildings, which probably served as elite residential-administrative buildings, were classified as domestic structures (Table 7). After calculating the rate of false positive and negative identifications based on gound-truthing data, we made some adjustments considering potential biases in ground-truthing data [22].

**Table 7:** Ground-truthing results by vegetation types.

Vegetation type	Classification	LiDAR analysis	Ground-truthing				Accuracy		Adjusted accuracy	
			Target verified	Positive verification	Discarded	Field IDed	Commission error	Omission error	Commission error	Omission error
High vegetation^a^	Structure	1,752	115	110	5	47	4.3%	29.9%	4.3%	29.9%
	Possible structure	1,232	18	16	2	0	11.1%		11.1%	
	Non-domestic	201	21	21	0	0	0.0%	0.0%		
Low vegetation^b^	Structure	2,185	178	173	5	32	2.8%	15.6%	2.8%	35.9%^d^
	Possible structure	1,271	24	15	9	0	37.5%		37.5%	
	Non-domestic	149	21	21	0	0	0.0%	0.0%		
Pasture	Structure	5,118	472	449	23	89	4.9%	16.5%	4.9%	16.5%
	Possible structure	1,878	72	46	26	0	36.1%		36.1%	
	Non-domestic	230	52	52	0	0	0.0%	0.0%		
Other^c^	Structure	549	4	4	0	7	0.0%	63.6%	4.3%^e^	19.2% ^e^
	Possible structure	157	1	1	0	0	0.0%		32.2% ^e^	
	Non-domestic	24	3	3	0	0	0.0%	0.0%		
Entire area	Structure	9,604	769	736	33	175	4.3%	19.2%		
	Possible structure	4,538	115	78	37	0	32.2%			
	Non-domestic	604	97	97	0	0	0.0%	0.0%		
Total		14,746	981	911	70	175				

^a^Includes rainforest, partially disturbed rain forest, high secondary vegetation, and partially cut high secondary vegetation.

^b^Includes medium-high secondary vegetation, low secondary vegetation, oil palm plantation, high grass, and *milpa* (agricultural field).

^c^Includes wetland forest and areas along the edges of the LiDAR-surveyed area where vegetation classification was not done.

^d^Because we could not obtain landowners’ permission to cut low vegetation, we suspect that substantial numbers of structures in these areas were overlooked during our ground-truthing [22]. We tentatively estimate that the omission error rate for this vegetation type is 20% higher than that for high vegetation.

^e^Since we do not have an enough number of ground-truthed samples for these areas, we applied the commission and omission rates from the entire Ceibal region.

By applying the adjusted commission and omission error rates listed in Table 7, we estimated structure frequencies, populations, and their densities by zones (Table 8). The use of structure counts visible on the surface is the method of population estimate most commonly used by Maya archaeologists [70]. In calculating the population of Ceibal, Tourtellot assumed that 85.7% of domestic structures were dwellings, and 4.37 persons lived in a dwelling on average (2.72 dwellings per unit and 11.9 persons per unit)[76]. We also followed Tourtellot’s assumption that 90% of all structures were occupied contemporaneously, although this rate may have been lower because the surface collection did not date structures in peripheral zones specifically to the Late or Terminal Classic period. At many other lowland Maya sites, including Tikal, the central Peten lake zone, the Belize Valley, and Nohmul, researchers found similar Late Classic population peaks, with 80 to 100% of mapped structures dating to this period [70]. In Table 8 the numbers calculated with these variables are presented as high estimates. However, the excavation data from rapidly abandoned structures at Aguateca, Guatemala, and Cerén, El Salvador, suggest the possibility that ratios of non-dwelling structures in each residential unit were considerably higher [78–80]. Based on Inomata’s research at Aguateca [81], we also calculated low estimates by applying 54% as the ratio of dwellings within domestic structures and the occupancy rate of 4.5 persons per dwelling.

**Table 8:** Estimates of structure frequencies, populations, and their densities.

Zone	Classification	LiDAR analysis					Structure estimate		Population estimate			
		High vegetation	Low vegetation	Pasture	Other	Total	Frequency^a^	Density (/km^2^)	Low		High	
									Population^b^	Density (km^2^)	Population^c^	Density (km^2^)
Ceibal center	Structure	831	98	189	0	1,118	1,932.6	357.2	4,227	781.3	6,514	1,204.1
	Possible structure	445	21	40	0	506						
	Non-domestic	113	11	12	0	136	136.0	25.1				
Ceibal horst	Structure	881	1,609	3,333	169	5,992	9,988.3	78.2	21,844	171.1	33,666	263.7
	Possible structure	709	1,117	1,512	81	3,419						
	Non-domestic	88	90	127	11	316	316.0	2.5				
Subín horst	Structure	36	459	1,512	346	2,353	3,118.1	115.4	6,819	252.4	10,510	389.0
	Possible structure	42	77	216	26	361						
	Non-domestic	0	41	87	13	141	141.0	5.2				
Other areas	Structure	4	19	84	34	141	341.4	1.1	747	2.4	1151	3.7
	Possible structure	36	56	110	50	252						
	Non-domestic	0	7	4	0	11	11.0	0.0				
Entire area	Structure	1,752	2,185	5,118	549	9,604	15,380.5	32.7	33,637	71.5	51,841	110.3
	Possible structure	1,232	1,271	1,878	157	4,538						
	Non-domestic	201	149	230	24	604	604.0	1.3				
Total		3,185	3,605	7,226	730	14,746	15,984.5	34.0				

^a^Estimated frequency of structures in each zone (see Table 5 for the formula). The total estimated frequency of “structures” is a sum of the estimated numbers for “structures” and “possible structures.” “Non-domestic” structures are mostly large buildings, and we assume that all of them were identified in LiDAR analysis.

^b^Estimated domestic structures x 54% x 90% x 4.5 persons.

^c^Estimated domestic structures x 85.7% x 90% x 4.37 persons.

**Fig 13:**
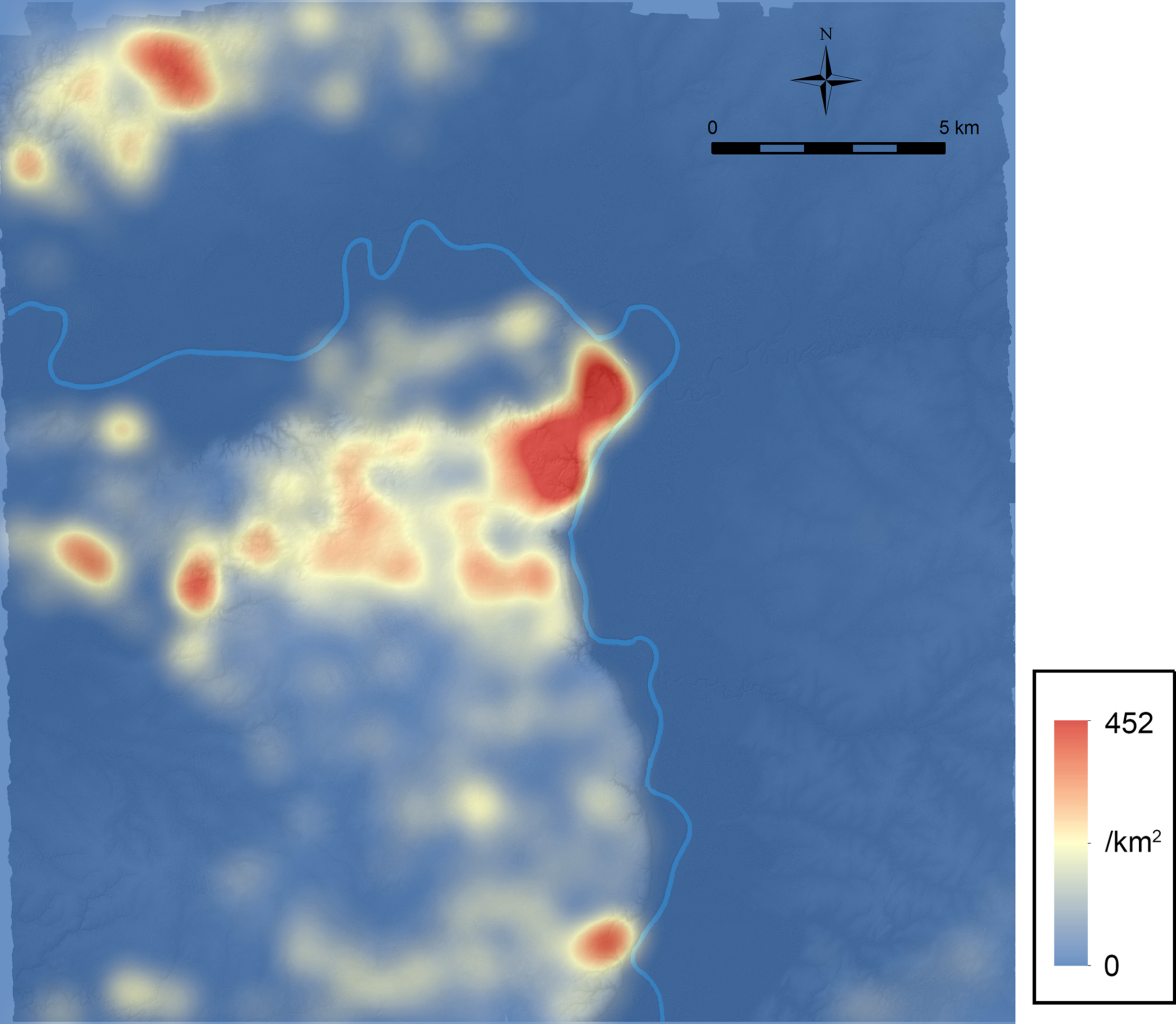
Structure density without non-domestic structures. It likely approximates the population distribution of the Late and Terminal Classic periods.

To visualize the distribution of population, we followed the same procedure used for Fig 10, using the counts of “structures” and “possible structures” obtained in LiDAR analysis, excluding non-domestic structures (Fig 13). We applied separate values of the variable [1-*C*_*g*_+*O*_*g*_(1-*C*_*g*_)/(1-*O*_*g*_)] for the population field for different vegetation types. Using the adjusted values in Table 7 as *C*_*g*_ and *O*_*g*_, we calculated the values for “structure” and “possible structure”: 1.365 and 0.889 for high vegetation, 1.517 and 0.625 for low vegetation, 1.140 and 0.639 for pasture, and 1.185 and 0.678 for other. Fig 13, as well as Table 8, shows the highest population concentration in the Ceibal center, but fairly high concentrations are also found outside the center, including: the areas around Anonal, El Edén, El Bramadero, El Cabro, El Cedral, and La Jutera. Although this population pattern generally correlates with the distribution of large units shown in Fig 11, comparison with the hot spot analysis (Fig 12) indicates some differences, as seen in the relatively low population density at the hot spot (a clustering of high-rank units) of Iberia. A possible explanation is that at Iberia the substantial construction mass of the Preclassic period may be misleadingly contributing to the high ranks of its units. Likewise, if the pyramidal complexes that were buried at the end of the Early Classic Junco 1 phase were not reused as ceremonial nodes during the Late and Terminal Classic periods, the high concentration of high-rank units in the Ceibal center may be slightly exaggerated. In contrast, the high population density areas southwest of Anonal, around El Bramadero, El Cabro, and El Cedral correspond to cold spots in Fig 12, meaning that these areas contain a concentration of low-rank units and smaller numbers of high-rank units. In these areas, Preclassic constructions of pyramidal complexes and platforms are scarce, which suggests that these communities developed mainly during the Late and Terminal Classic periods. If these patterns are correct, social inequality in these communities may have been less pronounced than at the center.

## Discussion

### Methodological issues

The results of our research shows the importance of vegetation classification and of evaluating LiDAR data by vegetation type. Omission errors in the identification of archaeological features, in particular, vary considerably between different land covers. Our research primarily employed a survey strategy of covering immediate vicinities of detected archaeological sites, as well as systematic coverages of three sample areas. The more extensive use of systematic coverage in different vegetation types would make a more rigorous evaluation of omission errors. In addition, we found the classification of “structures/supporting platforms” and “possible structures/supporting platforms” particularly effective. Because LiDAR detection errors occur mainly for small features measuring less than 50 cm in height, it is logical to separate features with low certainty of identification from those with higher certainty at the time of LiDAR analysis and to calculate their commission error rates separately after ground-truthing.

We developed the architectural chronology of our region based on the results of intensive excavations undertaken over 12 seasons by the CPAP and five seasons by the Harvard Project. We conducted the surface collection and test excavation programs in the LiDAR-surveyed area during the 2016 and 2017 to verify the applicability of this chronology to areas outside the Ceibal center. Our strategy of surface collection focusing on looters’ pit provided general chronological information on the Preclassic, Early Classic, and Late-Terminal Classic periods, although its results were not adequate for more precise dating within the Preclassic period. The results presented here need to be further examined with more extensive excavation programs in the future, but such operations over the extensive LiDAR-surveyed area will require significant time and resources. Collecting artifacts by scraping profiles of looters’ pits following stratigraphic layers may provide more detailed chronological data still fairly efficiently. Researchers working in areas without significant accumulations of archaeological data may still be able to make a tentative evaluation of diachronic patterns if applicable architectural chronologies are available from other areas. Our review of data showed a certain level of inter-regional consistency in Preclassic ceremonial complexes in the Maya lowlands, though some levels of variation are present.

### Diachronic process in the Ceibal region

During the early Middle Preclassic period (1000–700 BC), the E-Group assemblage in Ceibal Group A appears to have been a fixed locus for communal ceremony for the regional population that maintained varying levels of mobility (Fig 9A). We have argued elsewhere that the transition from the mobile lifeway to a more sedentary one and from a mixed subsistence economy to a heavier reliance on maize agriculture probably required substantial negotiation among diverse groups and individuals, particularly regarding changing notions of land and property ownership, community obligations, and increasing social inequality [82,83]. Public ceremonies and construction projects held at Ceibal probably offered opportunities for those groups to gather and to share common experiences [74,84,85].

The emergence of other E-Group assemblages and eastern winged pyramids, possibly from the early Middle Preclassic Escoba 2 phase to the Late Preclassic Cantutse 1 phase (600–300 BC), may signal the gradual acceptance of a more sedentary way of life (Fig 9B), although this assumption needs to be tested with more excavations, particularly of supporting platforms. As many groups began to settle down, the newly built ceremonial complexes may have marked the communal centers of smaller local groups, symbolizing their collective identities and their increasingly exclusive access rights to communal lands or their ownership. In excavations in the Ceibal center and at Caobal, remains of aquatic fauna, particularly shells, declined significantly around this period [86]. The development of communities in the inland part of the Ceibal horst implies that these groups relied primarily on maize agriculture, as opposed to aquatic resources of the river and wetlands, and that they secured their water sources during the dry season with the construction of small reservoirs. In the Ceibal horst, these ceremonial complexes were found within 8 km from Group A, and the Ceibal E Group was substantially larger than others. It is likely that Group A continued to serve as the primary ceremonial center where the regional population gathered on certain occasions. The presence of ceremonial complexes on the Subín horst, however, suggests that a separate network of ritual and political ties may have been developing. It is not clear whether the residents of the northern area participated in ceremonies held at Ceibal.

With the decline of La Venta and some Chiapas centers around 400 BC, Ceibal’s external relations changed, possibly leading to its political and ceremonial reorganization [37,87]. These changes are hinted by the emergence of pyramidal complexes that did not form E Groups or eastern winged pyramids over wide areas of the Ceibal region during the Late and Terminal Preclassic periods (350 BC-AD 175) (Fig 9C). If our dating of these ceremonial complexes is correct, their large number indicates that despite the political turmoil in southern Mesoamerica, Ceibal and associated polities continued to grow. The distribution of these complexes exhibits two trends. One is their spread into the peripheral zones of the horst, which probably indicates the expansion of populations into areas that were sparsely occupied in previous periods. The other trend is the high concentration of new complexes in the Ceibal center, which contrasts with the more even distribution of E Groups and winged pyramids in the preceding period. We can see a comparable concentration of platforms, which is suggestive of population distribution during the Late Preclassic period (Figs 9D and 10).

By the Late Preclassic period, a sedentary way of life was well established, and the period may be characterized by significant political centralization. Ceibal was certainly a prominent political center not only in the Ceibal region but also in the southwestern Maya lowlands. The intensive excavations of the CPAP show the emergence of similar ritual practices both in the Ceibal epicenter and in minor complexes with residential groups during the Late and Terminal Preclassic periods. These practices are represented by deposits of ceramic vessels and sacrificial burials, as well as the disappearance of figurines, which appear to have been tied to household ritual in previous periods [33]. We suspect that with the development of political centralization, the political and ritual symbolism tied to the central authority permeated through local groups and households [84,88].

The distribution of high-rank units during the Late and Terminal Classic periods (AD 600–830) exhibits their high concentration in the Ceibal center, comparable to that of pyramidal complexes possibly dating to the Late and Terminal Preclassic (Figs 11 and 12). The distribution of probable domestic structures of the Late and Terminal Classic periods also suggests a concentration of population in the Ceibal center, but it was not as pronounced as that of Preclassic platforms (Fig 13). The Preclassic platform density in the Ceibal center was 9.2 times higher than that of the rest of the Ceibal horst, and the population concentration in the Ceibal center might have been even greater if the population peak in the peripheral zones dates to a different period than that of the Ceibal center. The ratio for Classic-period structures was 4.6 (Tables 5 and 8). If our population estimates are reasonable, the peak population level of the Late and Terminal Classic periods was threefold to fivefold higher than that of the Late Preclassic period. Although Ceibal functioned as an important political and ceremonial center during the Late and Terminal Classic periods, the population appears to have been more spread out over the region.

Despite this population increase, our LiDAR analysis found few agricultural terraces, which contrasts with the high densities of terraces around Caracol, Belize [1]. The population in the Ceibal region, both during the Preclassic and Classic periods, concentrated in the uplands of the Ceibal horst and the Subín horst. The gently sloped areas between the uplands and the wetlands to the east and west of the Ceibal horst were largely unoccupied. Today’s farmers of the region use some of these areas for the cultivation of maize and other crops. With the wide availability of these agricultural lands in addition to cultivation in upland areas, the Prehispanic occupants of the Ceibal region may not have needed to construct agricultural terraces. It is not clear whether the Preclassic and Classic Maya did not live in the gently sloped area east of the Pasión River because they preferred better-drained uplands or because political and military competition between centers discouraged them from residing there.

## Conclusions

As areas of LiDAR survey rapidly expand, it is necessary to develop strategies to estimate diachronic processes on regional scales effectively and reasonably. Because LiDAR acquires detailed data on surface morphologies, an effective strategy may be to develop architectural chronologies based on morphological characteristics if the nature of archaeological sites is suitable. By emphasizing this approach, we do not mean to devalue the importance of intensive excavations. On the contrary, we maintain that detailed chronologies of occupation and construction can be obtained only through well-planned intensive excavations. Nonetheless, it is nearly impossible to conduct labor-intensive excavations throughout the often extensive area covered by LiDAR. As regional settlement data and associated archaeological information are always incomplete, a productive approach is to refine methods of extrapolation by combining the information on site morphologies derived from LiDAR with information from existing excavations, as well as data from efficient ground-truthing, surface collection, and test excavations.

Our reconstruction of diachronic processes in the Ceibal region needs to be further tested and refined through more excavations, test-pits, and surface collections. In particular, the possibility of intra-region variation in the architectural chronology should be examined by future excavations. Despite this qualification, the architectural chronology presented here shows a certain level of consistency across different regions of the Maya lowlands, indicating its validity within the recognized range of error. In the Ceibal region, we estimated a diachronic process by examining shifts in architectural format from the La Venta-type E Group to the Cenote-type E Group and eastern winged pyramids, and then to other forms of pyramidal complexes over the course of the Preclassic period. In addition, we reconstructed diachronic change in settlement pattern by assuming the transition in the configuration of residential groups from supporting platforms in the Preclassic to individual structures surrounding patios in the Classic. Test excavations and surface collection in sampled areas also provided additional evidence to support the applicability of this chronology. If our chronological reconstruction is correct, Ceibal served as the primary ceremonial complex for communal gatherings in a gradual transition from mobile to sedentary lifeways during the early Middle Preclassic period (1000–700 BC). With the development of sedentism and increasing reliance on maize agriculture, other ceremonial complexes were probably built, possibly symbolizing local groups’ claims to surrounding lands. During the following Late and Terminal Preclassic periods (350 BC-AD 175), ceremonial complexes and population appear to have expanded over a wider area while exhibiting a greater concentration in the Ceibal center. After the population decline at the beginning of the Early Classic period, the Ceibal region regained its political and economic vigor during the Late and Terminal Classic periods (AD 600–950) to support an even larger population. Settlements, however, concentrated on uplands, and extensive areas of gentle slopes between the uplands and wetlands remained nearly unoccupied.
